# Chromene-Thiazole Derivatives as Potential SARS-CoV‑2 M^pro^ Inhibitors: Synthesis and Computational Studies

**DOI:** 10.1021/acsomega.5c07593

**Published:** 2025-12-17

**Authors:** Lauren D. Stettler, Vincent T. Kopysciansky, Jenna E. Poor, Gabriela de Lima Menezes, Elton VanNoy, Guilherme Bastos Alves, Blake M. Shellenberger, Faith Garasich, Sylvia Stanell, Katyanna Sales Bezerra, Jonas Ivan Nobre Oliveira, Umberto Laino Fulco, Geneive E. Henry

**Affiliations:** † Department of Chemistry, 7296 Susquehanna University, 514 University Avenue, Selinsgrove, Pennsylvania 17870, United States; ‡ Bioinformatics Multidisciplinary Environment, Programa de Pós Graduação em Bioinformática, 28123 Universidade Federal do Rio Grande do Norte, Natal 59078-400, RN, Brazil; § Departamento de Biofísica e Farmacologia, Universidade Federal do Rio Grande do Norte, 59072-970 Natal-RN, Brazil

## Abstract

Three chromene-thiazole derivatives bearing benzimidazole, benzothiazole, and phenyl-1,2,4-triazole moieties were synthesized and evaluated for their potential as SARS-CoV-2 M^pro^ inhibitors. The derivatives were characterized by various spectroscopic and spectrometric methods: FT-IR, ^1^H NMR, ^13^C NMR, HRMS. Density functional theory (DFT) at the B3LYP/6–311++G­(3df,3pd) level was used to calculate the optimized structures of the derivatives and determine their electronic properties. Molecular docking analyses of the derivatives with SARS-CoV-2 M^pro^ (PDB ID: 6LU7) indicate significant interactions, with docking affinity scores ranging from −7.5 kcal/mol for the benzothiazole derivative to −8.4 kcal/mol for the phenyl-1,2,4-triazole derivative. These docking scores are comparable to or better than those of ML188 (−7.5 kcal/mol), a potent SARS-CoV-2 M^pro^ inhibitor, indicating the inhibitory potential of these derivatives. Molecular dynamics simulations and QM/MM calculations of the derivatives confirmed the stability of the protein–ligand interactions, and highlighted the key amino acid residues involved in stabilization.

## Graphical abstract

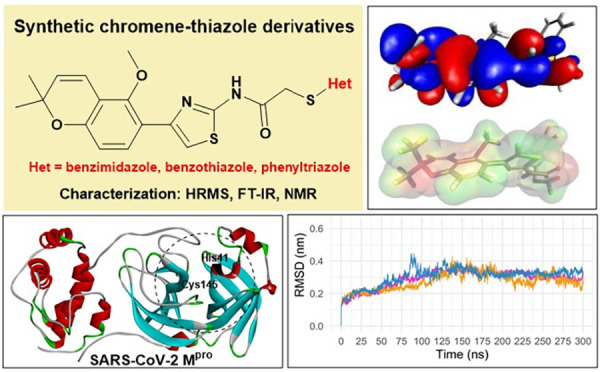

## 1 Introduction

The COVID-19 disease outbreak in 2019 quickly developed into a pandemic, and has resulted in over 778 million confirmed cases, including 7 million deaths globally.^1^ The disease is caused by the severe acute respiratory syndrome coronavirus-2 (SARS-CoV-2).^2–6^ The virus is a highly transmissible RNA virus that mutated rapidly during the early stages of the pandemic. The genome of the virus was solved quickly, and several proteins were identified as essential for host infection and subsequent viral replication. These include the structural spike protein and the nonstructural proteins, main protease (M^pro^), papain-like protease (PL^pro^) and RNA-dependent RNA polymerase (RdRp).^6–9^ The spike protein on the surface of the virus recognizes and binds to angiotensin-converting enzyme 2 (ACE2) receptor on the host cell surface, which sets in motion a cascade of events that lead to entry of the virus into the host cell. Inside the cell, M^pro^ and PL^pro^ are primarily responsible for viral replication, while the RdRp is essential for both viral replication and genome transcription.^6–9^ The search for new chemical inhibitors to target these proteins and others, simultaneous with the development of vaccines and antibodies, began at the outset of the pandemic. The first vaccines were developed within a year, which helped to reduce infection rates and control the spread of the disease.^7–9^ Subsequently, Paxlovid (Nirmatrelvir/Rotinavir), a M^pro^ inhibitor, received FDA approval as the first oral COVID-19 treatment.^10,11^ SARS-CoV-2 continues to mutate and remains a public health threat. Thus, continued development of new therapies is needed.

The M^pro^ remains one of the most widely studied SARS-CoV-2 targets, owing to its important role in the viral life cycle. The enzyme is involved in the hydrolysis of the viral replicase polyproteins (pp1a and pp1ab) at distinct locations to produce nonstructural proteins that are important for replication and transcription.^12–20^ The M^pro^ is a cysteine protease, which contains a His41-Cys145 catalytic dyad at the active site. The cysteine residue functions as a nucleophile in peptide bond cleavage, while the histidine residue is involved in acid–base catalysis.^12,14,15^ Covalent M^pro^ inhibitors such as Nirmatrelvir contain an electrophilic warhead that forms a covalent bond to the cysteine residue, thus inhibiting enzyme function.^10,11,21,22^ However, a major drawback of covalent inhibitors is that they are nonselective and are known to target other proteases such as calpain 1, trypsin, and cathepsins L and K.^23^ Conversely, noncovalent inhibitors such as ML188 affect M^pro^ function by forming intermolecular interactions with active site amino acid residues, offering greater selectivity over covalent inhibitors.^21,24^ Together with His41 and Cys145, key M^pro^ active site residues include Leu27, Met49, Phe140, Leu141, Gly143, His163, Met165, Glu166, Pro168, Gln189, Thr190 and Gln192. In the search for effective noncovalent M^pro^ inhibitors, computational methods, including molecular docking and molecular dynamics simulations, have become important tools to probe intermolecular interactions between M^pro^ amino acid residues and chemical entities.^25–28^

Despite a wide variety of existing chemical scaffolds as M^pro^ inhibitors, ongoing discovery and development of new scaffolds for the treatment of COVID-19 is important. Five-membered and six-membered heterocycles containing N, O and S, such as chromene, thiazole, imidazole, and triazole, are important pharmacophores for a number of disease classes. As a result, they are increasingly being explored as noncovalent M^pro^ inhibitors.^29–32^

The 2*H*-chromene moiety (Figure 1A), containing a six-membered oxygen heterocycle, has been explored as a scaffold for M^pro^ inhibition, using both experimental and theoretical methods.^33–35^ We have recently reported the M^pro^ inhibitory activity of furanochromene quinoline hydrazone (**I**),^33^ which shows good in vitro activity (IC_50_ = 16 μM), with strong docking affinity. Tuaimenal A (**II**), a natural product, showed comparable inhibition of M^pro^ (IC_50_ = 21 μM) to that of compound **I**, and also displayed favorable docking affinity.^34^ Furthermore, a series of chromene-containing carbazole derivatives, including koenine (**III**), was explored as M^pro^ inhibitors by computational methods and showed promising results.^35^

**Figure 1:**
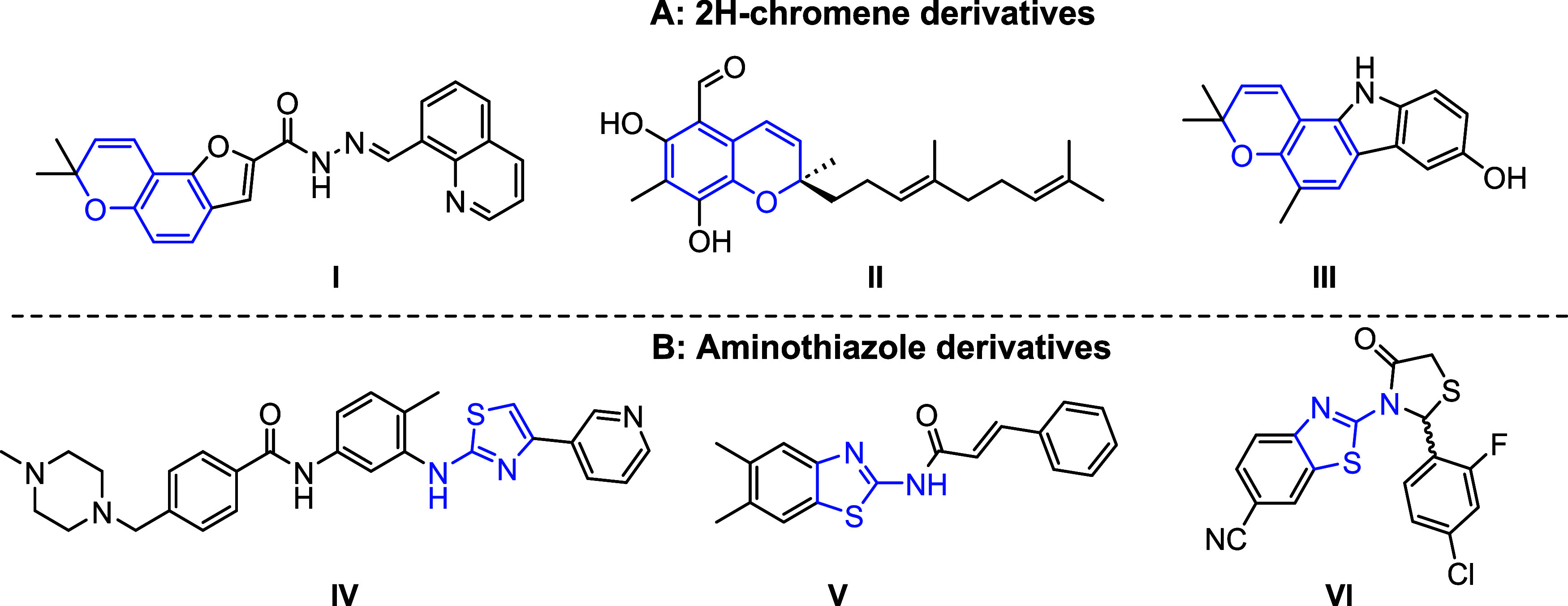
2*H*-chromene and aminothiazole derivatives as noncovalent SARS-CoV-2 M^pro^ inhibitors.

The thiazole moiety, containing nitrogen and sulfur, has also emerged as a promising scaffold for the development of M^pro^ inhibitors. Similar to the chromene moiety, several thiazole derivatives have demonstrated significant in vitro M^pro^ inhibitory activity and/or good docking affinity through molecular docking studies.^36–46^ Three of these derivatives are illustrated in Figure 1B. Masitinib (**IV**), an aminothiazole-based tyrosine kinase inhibitor, has been reported to be a potent noncovalent M^pro^ inhibitor (IC_50_ = 3 μM), and has also inhibited viral replication in cultured cells.^41^ The *N*-acyl aminothiazole derivative (**V**) also showed significant inhibition of M^pro^ (IC_50_ = 15 μM),^42^ while the thiazolidinone derivative (**VI**) was very potent (IC_50_ = 10 nM).^43^ In addition to their in vitro effects, these three thiazole derivatives also exhibited favorable docking affinity to the M^pro^ enzyme.

Five-membered nitrogen containing aromatic heterocycles such as imidazole and 1,2,4-triazole have been studied as M^pro^ inhibitors, and show similar activity profiles and docking interactions to those of the chromene and thiazole moieties.^40,45–50^ Molecular hybridization, which involves the combination of two or more pharmacophoric units, has been a useful strategy in the design of M^pro^ inhibitors.^51^ The pharmacophoric units may be merged, fused or separated by a linker, which in many cases also displays biological activity. Owing to the need for continued development of new M^pro^ inhibitors, we have designed a core chromene-thiazole scaffold, in which the two moieties are linked via a single bond. Additionally, three derivatives were created, in which the chromene-thiazole core is linked to benzimidazole, benzothiazole, and phenyl-1,2,4-triazole units. The benzothiazole is a bioisostere of benzimidazole, while the phenyl-1,2,4-triazole differs from benzimidazole by the addition of a nitrogen atom in the 5-membered ring and the incorporation of a pendant phenyl group instead of the fused ring system. The chromene-thiazole core is linked to the pendant heterocyclic units via a thioacetamido linker, which is present in several antiviral agents.^52^ The thioacetamido linker has also been used in the design of effective M^pro^ inhibitors, incorporating the molecular hybridization strategy.^50,52^

Herein, we describe the design, synthesis and characterization of these chromene-thiazole derivatives and exploration of their electronic properties using density functional theory (DFT) calculations. Molecular docking and molecular dynamics simulations, together with quantum mechanical calculations, were used to investigate the interaction of the derivatives with amino acid residues in the M^pro^ active site, and explore structure-affinity correlations.

## 2 Experimental Section

### 2.1 Chemicals and Instrumentation

Chemicals and solvents were purchased from Sigma-Aldrich, TCI America, or Fisher Scientific. Reactions were monitored by TLC analysis using silica gel plates. Column chromatography was performed on a Teledyne CombiFlash Rf 200 system, using RediSep Gold normal phase and reversed-phase silica gel columns. Melting points were recorded on a Thomas-Hoover capillary melting point instrument. High resolution mass spectrometry data were acquired using an Agilent 6560 ion mobility Q-ToF mass spectrometer with an Agilent Jet Spray dual ESI inlet (NSF MRI: CHE-2018547). Samples were run in positive mode by flow injection analysis in LC-MS grade 50% acetonitrile and 50% water containing 0.10% formic acid. FTIR data were acquired on a Nicolet iS50 spectrometer, with attached attenuated total reflectance (ATR) apparatus. NMR data (^1^H, 400 MHz and ^13^C, 100 MHz) were obtained using a JEOL 400 MHz instrument (NSF MRI: CHE-1625340).

### 2.2 Synthesis

Solvent gradients and column information for the purification of all compounds are displayed in the Supporting Information (Table S1).

#### 2.2.1 1-(5-hydroxy-2,2-dimethyl-2*H*-chromen-6-yl)­ethan-1-one (1)

A solution of 2,4-dihydroxyacetophenone (12.05 g, 79.2 mmol), 3-methyl-2-butenal (15.14 mL, 158.4 mmol) and pyridine (6.41 mL, 79.2 mmol) was heated under reflux for 24 h, followed by removal of excess reagents *in vacuo*. The residue was purified by column chromatography, eluting with 5% EtOAc-hexanes (isocratic), to give compound **1**.

Pale yellow solid (10.62 g, 61% yield, R_f_ = 0.67 in 30% EtOAc-hexanes).

The spectroscopic and analytical data for compound **1** were in agreement with the literature data.^53^

#### 2.2.2 1-(5-methoxy-2,2-dimethyl-2*H*-chromen-6-yl)­ethan-1-one (2)

A mixture of chromene **1** (11.15 g, 51.1 mmol), iodomethane (4.77 mL, 76.6 mmol), and cesium carbonate (33.29 g, 102 mmol) in dry DMF (80 mL) was stirred at room temperature for 24 h. The mixture was diluted with water (400 mL), followed by extraction with EtOAc (3 × 150 mL). The combined EtOAc solution was washed with saturated NaCl (200 mL), dried with Na_2_SO_4_, filtered and concentrated. Chromatography of the crude product using 5% EtOAc-hexanes (isocratic) gave compound **2**.

Yellow oil (10.47 g, 88% yield, R_f_ = 0.53 in 30% EtOAc-hexanes).

The spectroscopic and analytical data for compound **2** are in agreement with the literature data.^54^

#### 2.2.3 4-(5-methoxy-2,2-dimethyl-2*H*-chromen-6-yl)­thiazol-2-amine (3)

A mixture of chromene **2** (6.85 g, 31.4 mmol), iodine (8.76 g, 34.5 mmol) and copper­(II) oxide (2.75 g, 34.5 mmol) in absolute ethanol (200 mL) was heated under reflux. TLC analysis of the mixture after 1.5 h revealed the disappearance of the starting material. Thiourea (3.58 g, 47.0 mmol) was added and reflux was continued for 2 h, followed by the removal of ethanol under reduced pressure. Saturated NaHCO_3_ (200 mL) was added to the residue, followed by extraction with EtOAc (3 × 150 mL). The combined EtOAc solution was washed with 10% Na_2_S_2_O_3_ (200 mL) and dried over Na_2_SO_4_. After filtration, the extract was concentrated in vacuo. The residue was purified by column chromatography with 20% EtOAc-hexanes (isocratic) as eluent to give compound **3**.

Pale yellow solid (3.95 g, 44% yield, R_f_ = 0.18 in 30% EtOAc-hexanes); Mp 130–133 °C; IR (ATR), cm^–1^: 3405 (NH), 3370 (NH), 1634 (C=N), 1559 (Ar C=C).

^1^H NMR (DMSO-*d*_6_, 400 MHz) δ (ppm): 7.66 (1H, d, *J* = 8.4 Hz), 6.91 (2H, broad s, NH_2_), 6.89 (1H, s), 6.56 (1H, d, *J* = 10.0 Hz), 6.54 (1H, d, *J* = 8.4 Hz), 5.77 (1H, d, *J* = 10.0 Hz), 3.60 (3H, s), 1.34 (6H, s). ^13^C NMR (DMSO-*d*_6,_ 100 MHz) δ (ppm): 167.1, 153.8, 153.0, 146.1, 131.5, 130.1, 121.2, 117.1, 115.2, 112.5, 103.5, 76.4, 61.3, 28.0.

HRMS­(ESI): *m*/*z* calcd for C_15_H_17_N_2_O_2_S [M + H]^+^ 289.1011, found 289.1024.

#### 2.2.4 Synthesis of 2-chloro-*N*-(4-(5-methoxy-2,2-dimethyl-2*H*-chromen-6-yl)­thiazol-2-yl)­acetamide (4)

Chloroacetyl chloride (0.85 mL, 10.7 mol) was slowly added to a solution of aminothiazole **3** (2.35 g, 8.1 mmol), and triethylamine (1.13 mL, 8.1 mmol) in CH_2_Cl_2_ (20 mL) at 0 °C. The solution was slowly warmed to room temperature, and stirred for 18 h, followed by the addition of water (50 mL). The layers were separated and the aqueous phase was extracted with CH_2_Cl_2_ (2 × 20 mL). The combined CH_2_Cl_2_ solution was dried over Na_2_SO_4_, filtered and concentrated. The resulting brown oil was purified by column chromatography using 15% EtOAc-hexanes (isocratic) to give compound **4**.

Pale yellow solid (2.38 g, 80% yield, R_f_ = 0.49 in 30% EtOAc-hexanes); Mp 152–154 °C; IR (ATR), cm^–1^: 3370 (NH), 1656 (C=O), 1634 (C=N), 1574 (Ar C=C).

^1^H NMR (DMSO-*d*_6_, 400 MHz) δ (ppm): 12.52 (1H, s, NH), 7.69 (1H, d, *J* = 8.4 Hz), 7.50 (1H, s), 6.61 (1H, d, *J* = 8.4 Hz), 6.59 (1H, d, *J* = 10.0 Hz), 5.80 (1H, d, *J* = 10.0 Hz), 4.37 (2H, s), 3.62 (3H, s), 1.36 (6H, s). ^13^C NMR (DMSO-*d*_6,_ 100 MHz) δ (ppm): 165.5, 156.6, 154.1, 153.7, 145.5, 131.7, 129.8, 120.6, 116.9, 115.4, 112.8, 110.5, 76.6, 61.6, 42.9, 28.0.

HRMS­(ESI): *m*/*z* calcd for C_17_H_18_ClN_2_O_3_S [M + H]^+^ 365.0727, found 365.0718.

#### 2.2.5 General Synthesis for Heterocyclic Chromene-Thiazole Derivatives (**5–7**)

A mixture of chloroacetamide derivative (0.41−0.42 mmol), potassium iodide (1.2 equiv), potassium carbonate (1.5 equiv), and heterocyclic thiol (1.05 equiv) in acetonitrile (15 mL) was stirred at room temperature for 24 h. *For compound* **7** *, DMSO (1 mL) was added to the acetonitrile to aid in solubility of the thiol*. The acetonitrile was removed under reduced pressure. Water (15 mL) was added to the residue, and the aqueous solution was extracted with CH_2_Cl_2_ (3 × 10 mL). The combined CH_2_Cl_2_ solution was dried with anhydrous Na_2_SO_4_, filtered and concentrated. The resulting residue was purified by reversed-phase column chromatography. Compounds **5** and **7** were eluted with 75% MeOH/water, while compound **6** was eluted with 80% MeOH/water. The chromene-thiazole derivatives were characterized by IR, ^1^H NMR and ^13^C NMR spectroscopy, together with melting point and HRMS analyses.

##### 2.2.5.1. 2-((1*H*-Benzo­[*d*]­imidazol-2-yl)­thio)-*N*-(4-(5-methoxy-2,2-dimethyl-2*H*-chromen-6-yl)­thiazol-2-yl)­acetamide (**5**)

White solid (138 mg, 69% yield, R_f_ = 0.12 in 30% EtOAc-hexanes); Mp 120–123 °C; IR (ATR), cm^–1^: 3188 (NH), 1659 (C=O), 1632 (C=N), 1562 (Ar C=C).

^1^H NMR (DMSO-*d*_6_, 400 MHz) δ (ppm): 12.51 (NH), 7.71 (1H, d, *J* = 8.4 Hz), 7.45 (1H, s), 7.41 (2H, dd, *J* = 6.0, 3.6 Hz), 7.09 (2H, dd, *J* = 6.0, 3.6 Hz), 6.62 (1H, d, *J* = 8.4 Hz), 6.58 (1H, d, *J* = 10.0 Hz), 5.79 (1H, d, *J* = 10.0 Hz), 4.34 (2H, s), 3.61 (3H, s), 1.36 (6H, s). ^13^C NMR (DMSO-*d*_6,_ 100 MHz) δ (ppm): 167.2, 156.9, 154.1, 153.6, 149.9, 145.3, 131.7, 129.8, 122.1, 120.6, 116.9, 115.4, 112.8, 110.2, 76.5, 61.6, 35.3, 28.0.

HRMS­(ESI): *m*/*z* calcd for C_24_H_23_N_4_O_3_S_2_ [M + H]^+^ 479.1212, found 479.1202.

##### 2.2.5.2. 2-(Benzo­[*d*]­thiazol-2-ylthio)-*N*-(4-(5-methoxy-2,2-dimethyl-2*H*-chromen-6-yl)­thiazol-2-yl)­acetamide (**6**)

White solid (102 mg, 49% yield, R_f_ = 0.47 in 30% EtOAc-hexanes); Mp 188–190 °C; IR (ATR), cm^–1^: 3136 (NH), 1686 (C=O), 1633 (C=N), 1558 (Ar C=C).

^1^H NMR (DMSO-*d*_6_, 400 MHz) δ (ppm): 12.59 (NH), 8.00 (1H, d, *J* = 7.6 Hz), 7.78 (1H, d, *J* = 7.6 Hz), 7.72 (1H, d, *J* = 8.4 Hz), 7.48 (1H, s), 7.42 (1H, t, *J* = 7.6 Hz), 7.33 (1H, t, *J* = 7.6 Hz), 6.63 (1H, d, *J* = 8.4 Hz), 6.59 (1H, d, *J* = 10.0 Hz), 5.81 (1H, d, *J* = 10.0 Hz), 4.46 (2H, s), 3.62 (3H, s), 1.36 (6H, s). ^13^C NMR (DMSO-*d*_6,_ 100 MHz) δ (ppm): 166.4, 166.2, 156.8, 154.1, 153.7, 153.0, 145.4, 135.4, 131.7, 129.8, 127.0, 125.2, 122.5, 121.7, 120.6, 117.0, 115.4, 112.8, 110.3, 76.6, 61.6, 36.8, 28.0.

HRMS (ESI): *m*/*z* calcd for C_24_H_22_N_3_O_3_S_3_ [M + H]^+^ 496.0823; found 496.0813.

##### 2.2.5.3. *N*-(4-(5-methoxy-2,2-dimethyl-2*H*-chromen-6-yl)­thiazol-2-yl)-2-((5-phenyl-4*H*-1,2,4-triazol-3-yl)­thio)­acetamide (**7**)

White solid (160 mg, 76% yield, R_f_ = 0.16 in 30% EtOAc-hexanes); Mp 121–123 °C; IR (ATR), cm^–1^: 3446 (NH), 3164 (NH), 1674 (C=O), 1633 (C=N), 1561 (Ar C=C).

^1^H NMR (DMSO-*d*_6_, 400 MHz) δ (ppm): 14.44 (NH), 12.54 (NH), 7.92 (2H, d, *J* = 6.8 Hz), 7.72 (1H, d, *J* = 8.4 Hz), 7.46 (1H, s), 7.44 (3H, m), 6.62 (1H, d, *J* = 8.4 Hz), 6.59 (1H, d, *J* = 10.0 Hz), 5.80 (1H, d, *J* = 10.0 Hz), 4.18 (2H, s), 3.61 (3H, s), 1.36 (6H, s). ^13^C NMR (DMSO-*d*_6,_ 100 MHz) δ (ppm): 167.5, 157.0, 154.1, 153.6, 145.3, 131.7, 130.7, 129.8, 129.5, 126.5, 120.7, 117.0, 115.4, 112.7, 110.2, 76.5, 61.6, 35.5, 28.0.

HRMS (ESI): *m*/*z* calcd for C_25_H_24_N_5_O_3_S_2_ [M + H]^+^ 506.132058; found 506.1310.

### 2.3 Computational Analyses

#### 2.3.1 Ligand Optimization using DFT Calculations

A Dreiding-like force field was used to optimize the chromene-thiazole heterocyclic derivatives for DFT calculations. Two-phase minimization of the ligands was then performed using CHARMm and SmartMinimizer within a solvent environment represented by distance-dependent dielectrics. This was all implemented using the Discovery Studio software suite (https://www.3ds.com/products/biovia/discovery-studio). The Generate Conformations function and the ″BEST″ method were used to explore the conformations of the compounds, which ensured that the entire conformational landscape was examined. This method applies energy minimization techniques in both torsional and Cartesian space using the Poling algorithm with a stepwise process: first a conjugate gradient minimization in torsional space, followed by a similar minimization in Cartesian space, and finally a quasi-Newton minimization in Cartesian space.

To ensure greater accuracy, each conformation was carefully analyzed. To this end, systematic and stochastic torsion angle sampling methods were used together with the Boltzmann jump technique to evaluate conformational changes within predetermined energy thresholds, resulting in a wide range of stable molecular conformations.

In order to achieve robust geometry optimization and electronic characterization, quantum chemical calculations were performed using both molecular orbital theory (MO) and density functional theory (DFT). For these calculations, the 6–311++G­(3df,3pd) basis set was used, which contains the correct polarization and diffusion functions.^55,56^ The quantum descriptors evaluated included the energies of the highest occupied molecular orbital (HOMO), the lowest unoccupied molecular orbital (LUMO), and the GAP energy (εHOMO - εLUMO).^57^

#### 2.3.2 Molecular Docking Analyses with SARS-CoV-2 M^pro^

Molecular docking analysis was used to study the interactions of the chromene-thiazole ligands with SARS CoV-2 M^pro^. The X-ray crystal structure of M^pro^ (PDB ID: 6LU7) bound to N3 (*N*-[(5-methylisoxazol-3-yl)­carbonyl]­alanyl-l-valyl-*N*∼1∼-((1R,2Z)-4-(benzyloxy)-4-oxo-1-{[(3R)-2-oxopyrrolidin-3-yl] methyl}­but-2-enyl)-l-leucinamide) inhibitor was obtained from the Protein Data Bank (http://www.rcsb.org/pdb). After removal of the inhibitor and water molecules, the protein was prepared for docking by adding polar hydrogens and Kollman charges using Python Molecular Viewer. The protein protonation state was determined using PropKa online server (https://www.ddl.unimi.it/vegaol/propka.htm). Avogadro was used to generate the structures of the chromene-thiazole ligands, followed by energy optimization using a UFF force field with steepest-descent algorithm. Open Babel software was used to add polar hydrogens to the ligands at physiological pH, and also to convert pdb files to pdbqt format. Docking analyses were carried out using AutoDock Tools version 1.5.6 and AutoDock Vina programs.^58^ The grid box was set using the following parameters: spacing = 0.375 Å, grid center (*x* = −10.88, *y* = 13.94, *z* = 68.21), dimension (*x* = 58, *y* = 68, *z* = 70), and included key active site amino acid residues.^59^ Nine conformational images were created for each ligand, with the exhaustiveness value set at 8. Random seed values for compounds **5**, **6** and **7** are −762719520, 3036648, and 191580080, respectively. Validation of the docking procedure was performed by removing the N3 inhibitor from M^pro^ active site and redocking it, using the same grid parameters used for the chromene-thiazole ligands. However, the exhaustiveness was set to 12 and 10 conformations were obtained. The experiment was performed 100 times (1000 conformations), and RMSD calculations were used to determine the redocked conformation that was the best fit to the native inhibitor. The output files for the lowest energy conformers of the ligands were exported to Biovia Discovery Studio Client for display of the 3D structure of the M^pro^-chromene-thiazole ligand complexes.

#### 2.3.3 Molecular Dynamics Simulation

MD simulations were conducted to assess the stability of the chromene-thiazole ligands in solution and to modify their conformation within the M^pro^ active site. For this purpose, three independent replicates of 300 ns were conducted for each system using the GROMACS 2023 program.^60^ First, the parameters of the ligands for the GROMACS configuration were generated using the ACPYPE server (www.bio2byte.be/acpype/), with the Gasteiger charge as the method and GAFF2 as the force field.^61^ Amberff99SB-ILDN was used as the force field for the protein, which is provided by GROMACS.

A cubic box was constructed for each system, and the TIP3P water model was included to ensure that the minimum distance between the surface of the box and the solute (protease complex) was 12 Å. Furthermore, the systems were neutralized by adding Na^+^ ions. Two iterations of energy minimization (EM) were conducted to eliminate unfavorable contacts with the initial structure.

The initial EM was configured to operate for a maximum of 20,000 steps, or until the maximum force acting on each atom was less than 50 kJ/mol/nm. The protein and ligand were constrained in their positions to ensure that the EM focused on the solvent’s relaxation. In the second EM, where the coordinates of the protein and ligand were not constrained, a flexible solvent and the same algorithm were used. It was executed for a maximum of 10,000 steps or until the force applied to each atom was less than 250 kJ/mol/nm.

Using the modified Berendsen algorithm^62^ for temperature adjustment and control, the temperature of the system was maintained at 298 K for a duration of over 100 ps. The Parrinello–Rahman algorithm^63^ was used for the second stage, which involved pressure adjustment over 100 ps. Positional restraint of the protein–ligand complexes was implemented to stabilize the solvent surrounding the solute, and hydrogen atoms were constrained during both processes using the LINCS algorithm.^64^

The Particle Mesh Ewald (PME) summation method was employed to calculate long-range interactions, with a 1 nm cutoff for nonbonded interactions. The leapfrog algorithm^65^ was employed to integrate the equations of motion with a time-step of 2 fs. A brief 1 ns stage was conducted with the protein complex without positional restraint prior to the MD production stage, which was followed by a 300 ns production stage. A total of 3000 conformational frames were generated by each MD simulation. Root mean square deviation (RMSD) and fluctuation (RMSF) were calculated using the ″gmx″ commands of the GROMACS package.

To obtain the lowest energy complex from the MD simulations, hybrid Quantum Mechanics/Molecular Mechanics - Generalized-Born surface area (QM/MM-GBSA) calculations were performed using the gmx_MMPBSA program.^66^ For this, the last 50 ns (500 frames) of each MD simulation were used, since it is expected that the complex will be more stable by the end of the simulation. The QM region was restricted to residues that were within 5 Å of the ligand, and the semiempirical PM6-DH+ functional was implemented. For the remaining residues and for the ligands, the calculations were conducted using the same force field as in the MD. Explicit solvent molecules and ions were excluded from the analysis as GBSA method treats the solute as a low-dielectric region and the solvent as a high-dielectric continuum. The MFCC method was employed to select the lowest energy complexes for each of the systems for the QM calculations, which will be provided in the following section.

#### 2.3.4 Quantum-Mechanical Calculations Using Molecular Fractionation with Conjugated Caps

To obtain complete *ab initio* quantum mechanical results, the Molecular Fractionation with Conjugated Caps (MFCC) approach and Density Functional Theory (DFT)-based quantum calculations were used to accurately determine the protein–ligand interaction energy for each of chromene-thiazole derivatives.^67–69^ The MFCC method allows for accurate calculation of the interaction energies between proteins, DNA and other macromolecules, with high computational efficiency. It is based on the fragmentation of the biomolecule into smaller units, whose ends are adjusted with neighboring sections, maintaining the local context. This makes it possible to estimate protein–ligand interactions through sums of interactions between fragments, making it a robust tool to investigate complex biological systems.

Let R_*i*_ be the protein amino acid and *i*th residue that interacts with ligand L. In order to maintain the valencies and enhance the overall simulation of the actual electronic environment surrounding the interaction between R_*i*_ and L, conjugated caps *C*_*i*_ and *C*_i_^*^ are defined as the residues bond to R_*i*_’s amine and carboxyl group, respectively, with additional covalent bonds to hydrogen atoms in each extremity. For the N­(C)-terminal residue, cap *C*_*i*_(*C*_i_*) is empty.

With the aim of calculating the interaction energy (*IE*_*i*_) between the *L* and *R*_*i*_, we constructed four distinct subsystems, *A*_*i*_ = (*L* – *C*_*i*_ *R*_*i*_ *C*_i_*), *B*_*i*_ = (*C*_*i*_ *R*_*i*_ *C*_i_*), *C*_*i*_ = (*L* – *C*_*i*_ *C*_i_*) and *D*_*i*_ = (*C*_*i*_ *C*_i_*). Therefore,

1 $$IE_{i}=E\left(A_{i}\right)-E\left(B_{i}\right)-E\left(C_{i}\right)+E\left(D_{i}\right)$$

where *E*(*A*_*i*_), *E*(*B*_*i*_), *E*(*C*_*i*_) and *E*(*D*_*i*_) are the total energy values for each subsystem.

Energy calculations were carried out using the Gaussian G16 software after the MFCC fragmentation method was applied to each system. The DFT formalism, which is based on quantum mechanics (QM) and can accurately describe the intermolecular interaction energies at a computational cost that is reasonable, was used.^70^ The meta-generalized gradient approximation (meta-GGA) functional B97D was used for this work. Comparative experiments using various GGA functionals (including but not limited to ωB97XD, B3LYP, B97D, B97D3, M06, and M062-X) have demonstrated the effectiveness of B97D in determining the geometries of organic structures as well as the atomization and interaction energy.^71–73^ It also exhibits better thermochemical characteristics and energy calculations for noncovalent bound compounds; therefore, its advantageous to use because it requires little computing power and yields no unfavorable outcomes. In order to better depict the electronic wave function and enlarge the Kohn-Sahm orbitals, we used 6–311+G­(d,p), a triple split valence (triple-ζ) basis set that uses polarization functions (d,p) and an extra diffuse function (+).

Accurately simulating an appropriate electrostatic environment is crucial when researching biomolecular characteristics theoretically. This is accomplished by indirectly describing solvation effects through the simulation of an aqueous environment. The most popular technique for representing these effects at a manageable computational cost is the Self-Consistent Reaction Field (SCRF) method, which uses a Conductor-like Continuous Polarizable Model (C-PCM) with the formula SCRF = C-PCM. By putting the solute in a cavity with a dielectric constant, similar to that in vacuum circumstances inside and a desired solvent outside, it is possible to apply a continuum dielectric constant using this method.^74^

A medium with a lower permittivity is linked to a lower dielectric constant, which leads to an overestimation of interaction energies by permitting more energetic interactions; conversely, when the permittivity increases, the total energies tend to decrease. However, earlier research has indicated that a larger dielectric constant can be employed as a good compromise between models and experimental evidence.^75–77^ To simulate both a medium of lower permittivity that closely resembles the predicted mean dielectric constant of proteins and a higher value of ε that yields more accurate results in biological systems because it better represents the state of protein in a solvated state, values of ε = 10 and 40 were used in this context.

A convergence study was conducted to assess the total energy as a function of its distance as the radius increased, taking into account the spatial distribution of amino acid residues in the protein–ligand system. The amino acid residues within the binding pocket radius were analyzed by defining it as a distance of *r* = 2.0 to 10.0 Å with the ligand as its centroid. Subsequently, the total energy of each successive radius was observed by drawing imaginary circles with increments of *r* = 0.5 Å. Convergence is achieved when the total energy difference between a given radius and its consecutive is equal to or smaller than 10%. In other words, it was presumed that the most critical intermolecular interactions occurred up to the converging radius.^78,79^

## 3 Results and Discussion

### 3.1 Design and Synthesis of Chromene-Thiazole Derivatives

The new chromene-thiazole scaffold was designed based on evidence of chromene and thiazole moieties showing strong potential as M^pro^ inhibitors, evidenced by both experimental and computational studies.^31–35,41–43^ The rationale for incorporation of the benzimidazole, benzothiazole and phenyl-1,2,4-triazole groups is also based on studies indicating their potential as M^pro^ inhibitors.^45–50^ The benzothiazole is a bioisostere of benzimidazole, while the phenyl-1,2,4-triazole differs from benzimidazole by the addition of a nitrogen atom in the 5-membered ring and the incorporation of a pendant phenyl group instead of the fused ring system. The structural differences among these three heterocyclic scaffolds allows for examination of structure-reactivity and structure-docking affinity correlations.

The synthetic approach for the preparation of the heterocyclic chromene-thiazole derivatives (**5–7**) is depicted in Scheme 1. The chromene (**1**) was obtained in 61% yield by the reaction of 2,4-dihydroxyacetophenone and 3-methyl-2-butenal in pyridine.^53^ Using a modified version of a previously reported procedure,^54^ *O*-methylation of the chromene was achieved by reaction with iodomethane and cesium carbonate in DMF at room temperature to give compound **2** in 88% yield. The use of copper­(II) bromide for the α-bromination of ketones is well-documented.^80^ We envisioned using this method to generate the α-bromo ketone derivative of compound **2**, followed by condensation with thiourea to give the chromene-amino thiazole **3**. A similar strategy was recently used by Li et al.^81^ to assemble a chromene-thiazole derivative. However, in our hands, the yields were very low, partly because of hydrolysis of the methyl ether under the reaction conditions. Thus, the chromene-aminothiazole **3** was obtained in 44% yield via a two-step, one-pot reaction involving α-iodination in the presence of catalytic copper­(II) oxide, followed by addition of thiourea.^82^ Treatment of the aminothiazole with chloroacetyl chloride in the presence of triethylamine^83^ afforded the corresponding chloroacetamide **4** in 80% yield. The chloroacetamide was converted *in situ* to the corresponding iodide using potassium iodide, followed by nucleophilic substitution with the heterocyclic thiol under basic conditions to afford the benzimidazole (**5**), benzothiazole (**6**), and phenyl-1,2,4-triazole (**7**) derivatives in 49–76% yield.^84,85^

**Scheme 1:**
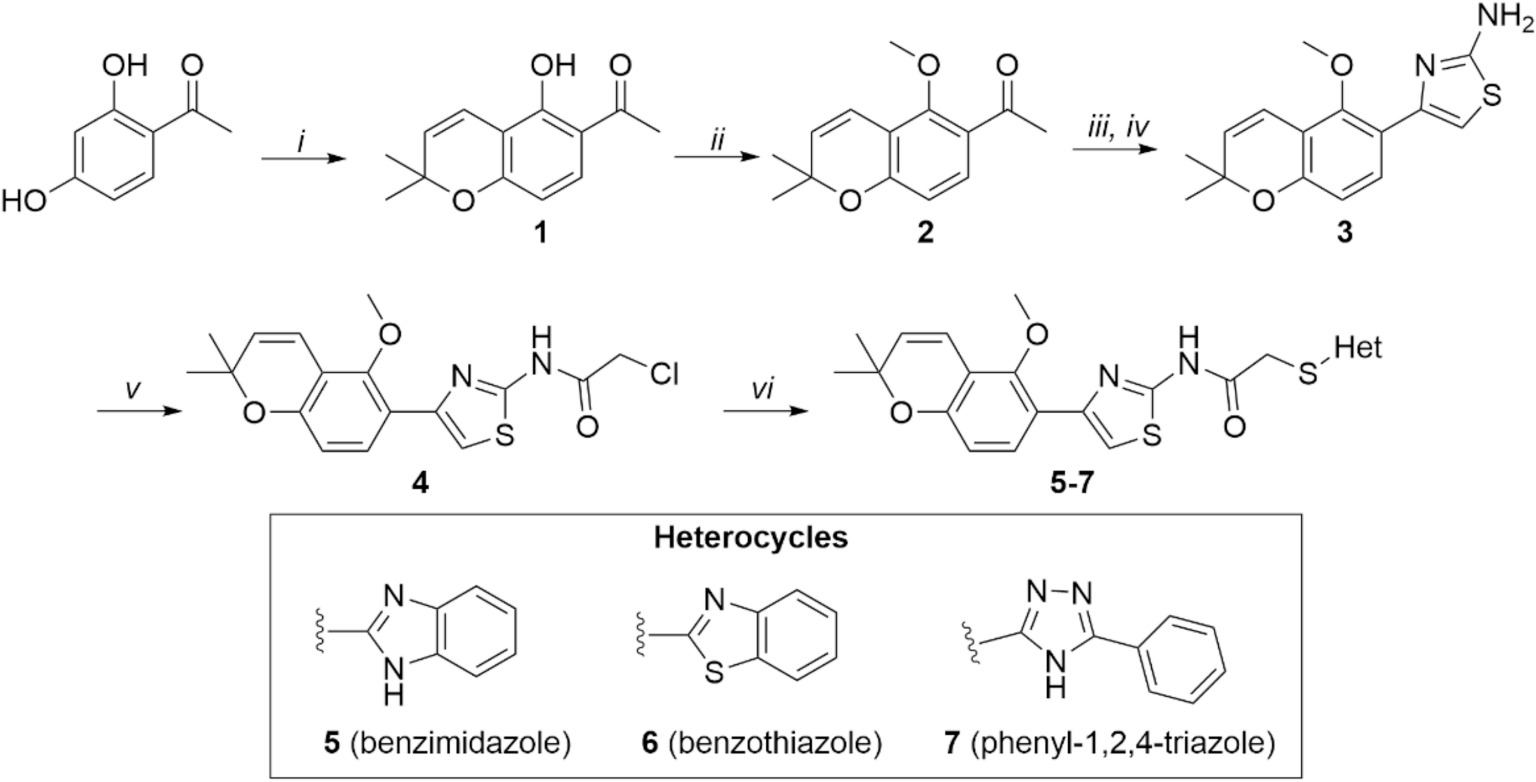
Synthesis of Chromene-Thiazole Heterocyclic Derivatives (**5–7**)­[Fn s1fn1]

a
*Reagents and conditions*: (i) 3-methyl-2-butenal, pyridine, heat, 24 h; (ii) CH_3_I, Cs_2_CO_3_, DMF, rt, 24 h; (iii) I_2_, CuO, EtOH, reflux 1.5 h; (iv) thiourea, EtOH, reflux, 2 h; (v) chloroacetyl chloride, triethylamine, CH_2_Cl_2_, rt, 18 h; (vi) appropriate heterocyclic thiol, KI, K_2_CO_3_, CH_3_CN, rt, 24 h.

### 3.2 Spectroscopic Characterization

The structures of the chromene-thiazole derivatives and precursors **1–7** were verified by spectral analyses. Data for compounds **1** and **2** have been previously reported.^53,54^ The high-resolution mass spectrometric data (positive ion mode) for compounds **3–7** were in good agreement with the calculated values (2.2.3–2.2.5; Figure S1–S5). The IR spectra of compounds **3** and **4** (Figures S6–S7) displayed characteristic NH signals between 3300 and 3500 cm^–1^ and peaks corresponding to the C=N and aromatic C=C bonds at 1634 and 1559–1574 cm^–1^, respectively.^82,83^ In addition, the peak for the C=O bond of compound **4** was observed at 1656 cm^–1^. The IR spectra of compounds **5–7** (Figures S8–S10) displayed NH, C=N and C=C absorption bands in similar ranges to those of compound **4**. The C–S stretches for the thioether, which appear in the fingerprint region, were not assigned owing to medium/low peak intensity and overlap. However, ^1^H and ^13^C NMR spectra (Figures S11–S20) in DMSO-*d*_6_ allowed for unambiguous characterization of the chromene-thiazoles.

Compound **3** displayed the characteristic ^1^H NMR signals for the dimethyl chromene moiety: doublets (*J* = 8.4 Hz) at 7.66 and 6.54 ppm, attributed to the aromatic protons; doublets (*J* = 10.0 Hz) at 6.56 and 5.77 ppm, assigned to the alkene protons; and a singlet at 1.34 ppm, attributed to the *gem*-dimethyl group. The signals for the protons in the amino group, thiazole ring and methoxy group appeared as singlets at 6.91, 6.89, and 3.60 ppm, respectively, with the appropriate integrations. The ^13^C NMR signals for the chromene and thiazole rings were in good agreement with expected values.^33,81^ The ^1^H NMR spectrum of compound **4** displayed one additional peak, relative to compound **3**. The α-methylene protons were observed as a singlet at 4.37 ppm. The amide NH was significantly deshielded, appearing at 12.52 ppm, which is characteristic of chloroacetamides.^83^ In addition, the proton of the thiazole ring was slightly deshielded, with a value of 7.50 ppm. The ^13^C NMR signal for the amide carbonyl was observed at 165.5 ppm, which is diagnostic for this functional group.^83^ For the heterocyclic derivatives (**5–7**), the ^1^H NMR signals for the chromene-thiazole ring showed similar splitting patterns and chemical shift values to those observed for compounds **3** and **4**. Chemical shift values for the α-methylene protons of compounds **5**, **6** and **7** were observed at 4.34, 4.46, and 4.18 ppm, respectively. The amide protons were observed in a similar range as compound **4**, with the peaks for compounds **5** and **7** being very broad. Chemical shift values for the heterocyclic unit connected to the thioacetamido linker were displayed in the predicted ranges.^84,85^ The protons on the *ortho*-disubstituted benzene ring of the benzimidazole derivative (**5**) were observed as two doublet of doublets at 7.41 and 7.09 ppm (*J* = 6.0, 3.6 Hz), with the equivalent peaks attributed to tautomerization.^86^ The corresponding protons for the benzothiazole derivative (**6**) were shown as doublets (*J* = 7.6 Hz) at 8.00 and 7.78 ppm, and triplets (*J* = 7.6 Hz) at 7.42 and 7.33 ppm. For the phenyl-1,2,4-triazole derivative (**7**), the NH peak for the triazole ring was observed at 14.44 ppm, while the signals for the phenyl ring were observed as a doublet (*J* = 6.8 Hz) and multiplet at 7.92 and 7.42 ppm, respectively. The ^13^C NMR spectra showed the expected signals for the derivatives, with the amide carbonyl occurring between 166 and 168 ppm.

### 3.3 Density Functional Theory (DFT) Studies

Electronic parameters play a key role in elucidating the interaction between organic ligands and amino acid residues in protein drug targets. Thus, DFT studies were performed to determine the electronic properties of the chromene-thiazole derivatives prior to molecular docking and molecular dynamics analyses with the M^pro^ enzyme.

#### 3.3.1 Frontier Molecular Orbitals and Chemical Reactivity Descriptors

Analysis of frontier molecular orbitals (FMOs) was performed to gain insight into the chemical reactivity of the chromene-thiazole derivatives (**5–7**). HOMO–LUMO diagrams of the derivatives are displayed in Figure 2, and HOMO, LUMO and band gap energy values are displayed in the Supporting Information (Table S2). The red and blue colors represent the positive and negatives phases of the HOMO–LUMO orbital, respectively.^87^ The general trend observed for the derivatives is that the HOMO orbitals are localized predominantly on the chromene-thiazole portion of the molecule. For the benzimidazole (**5**) and phenyl-1,2,4-triazole (**7**) derivatives, the LUMO orbitals are mainly distributed over the pendant heterocyclic system, while for the benzothiazole (**6**) derivative, they are localized over the two thiazole rings and the thioacetamido linker. The HOMO and LUMO energy values are directly correlated with the electron-donating and electron-accepting properties of molecules. Higher HOMO values signify greater electron-donating ability, while lower LUMO values indicate better electron-accepting tendency, and increased likelihood of undergoing electrophilic reactions. Additionally, HOMO–LUMO band gap energy, *E*_gap_ (*E*_LUMO_ – *E*_HOMO_), gives an indication of the chemical reactivity of the molecule, with a smaller value indicating higher chemical reactivity. The order of electron-donating ability of the derivatives is **5** > **6** > **7**, while the electron-accepting ability shows the reverse trend. Based on the *E*_gap_ values, the phenyl-1,2,4-triazole derivative (**7**) is most reactive, while the benzimidazole derivative (**5**) is least reactive. The lower *E*_gap_ for the phenyl-1,2,4-triazole derivative, combined with the greater electron-accepting ability, indicates potentially stronger interactions with electron-rich sites in biological targets.^40,87–89^

**Figure 2:**
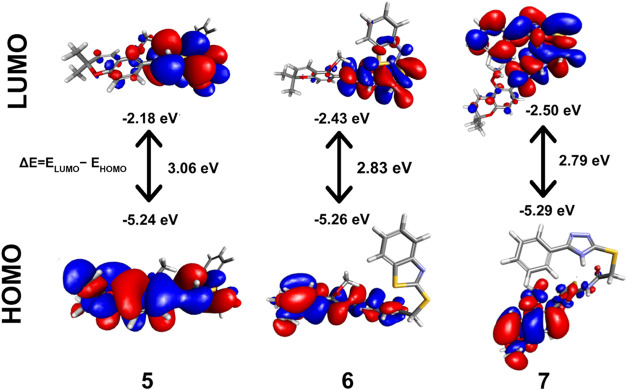
HOMO–LUMO and their energy gap for chromene-thiazole derivatives (**5–7**).

#### 3.3.2 Molecular Electrostatic Potential (MEP)

The molecular electrostatic potential (MEP) map is used to illustrate the charge distribution in a molecule. The MEP maps are complementary to the FMO diagrams and can also be used to predict interactions with biological targets. The red color (most negative potential) on the map represents the electron-rich sites, which are favored for electrophilic attack, while the blue color (most positive potential) represents electron-deficient regions which are susceptible to nucleophilic attack.^90^ The MEP map for the optimized structures of chromene-thiazole derivatives is displayed in Figure 3. For all three derivatives, the heterocyclic systems at the ends of the molecules (chromene and N/S heterocycles) have the most negative potential, which was pronounced around the nitrogen atoms of the imidazole, thiazole and triazole rings. These are potential sites for hydrogen bonding interactions with biological targets.^36,91,92^ Following the trend from the FMO analysis, the benzimidazole derivative (**5**) displays the least potential for nucleophilic attack, while the phenyl-1,2,4-triazole derivative (**7**) shows the greatest susceptibility for nucleophilic attack.

**Figure 3:**
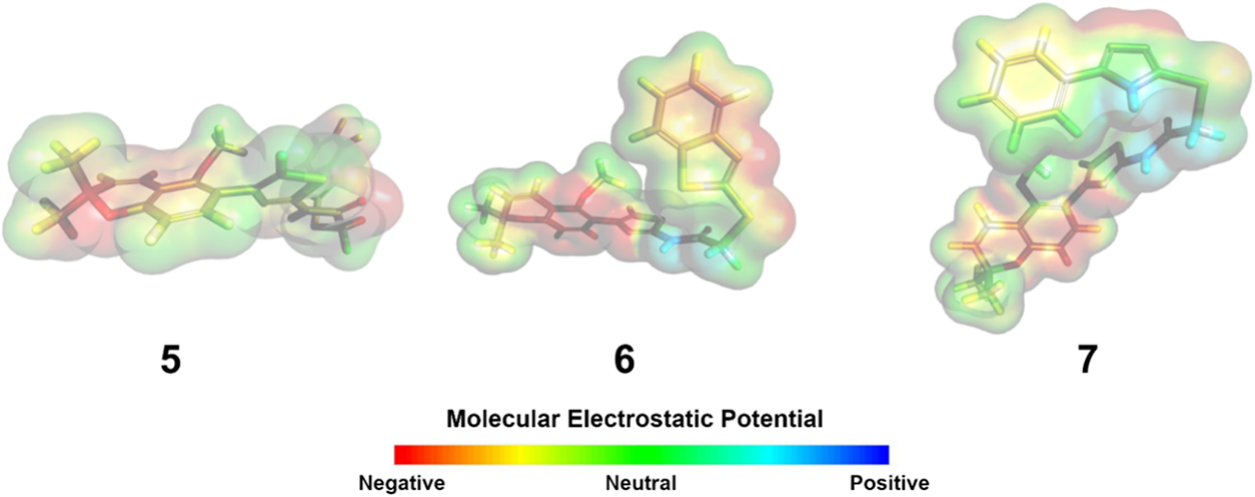
Molecular electrostatic potential (MEP) map for chromene-thiazole derivatives (**5–7**).

### 3.4 Molecular Docking Analysis with SARS-CoV-2 M^pro^

Based on the information gained from the FMO and MEP analyses of the chromene-thiazole derivatives, which predicts their interaction with biological targets, their potential to act as noncovalent M^pro^ inhibitors was evaluated by molecular docking analyses using AutoDock Vina. The M^pro^ protein (PDB ID: 6LU7) was kept rigid, while the ligands were flexible. Docking scores for the lowest energy binding pose for benzimidazole (**5**), benzothiazole (**6**) and phenyl-1,2,4-triazole (**7**) with M^pro^ were −7.8, −7.5, and −8.4 kcal/mol, respectively. Notably, the benzimidazole and phenyl-1,2,4-triazole derivatives showed better docking affinity relative to ML188 (−7.5 kcal/mol), a potent noncovalent inhibitor.^24^

Figure 4A shows the binding region of the M^pro^ enzyme and the location of the His41 and Cys145 that make up the catalytic dyad between domains I and II. The orientations of ML188 and the derivatives in the binding pocket are displayed in Figure 4B-4E. ML188 is positioned such that it interacts significantly with both domains I and II. The *tert*-butyl amide and furan groups are located close to His41 and Cys145, respectively, while the pyridine ring is situated between both amino acids. The benzimidazole derivative (**5**) adopts an orientation in which the chromene ring is in close proximity to domain II, with the *gem*-dimethyl group oriented toward the linker loop, and the benzimidazole group is close to domain I (Figure 4C). This orientation puts the majority of the molecule close to both the His41 and Cys145 residues. The benzothiazole derivative (**6**) adopts a pose in which the chromene moiety and benzothiazole moieties are oriented opposite of that observed for its bioisostere, compound **5** (Figure 4D). Similar to compound **5**, this orientation allows for close association with the His41 and Cys145 residues. For the phenyl-1,2,4-triazole derivative (**7**), the chromene ring sits higher in the binding pocket relative to that of the benzimidazole derivative, with reduced interaction with domain I. However, the phenyl-1,2,4-triazole moiety is closely associated with the His41 and Cys145 residues.

**Figure 4:**
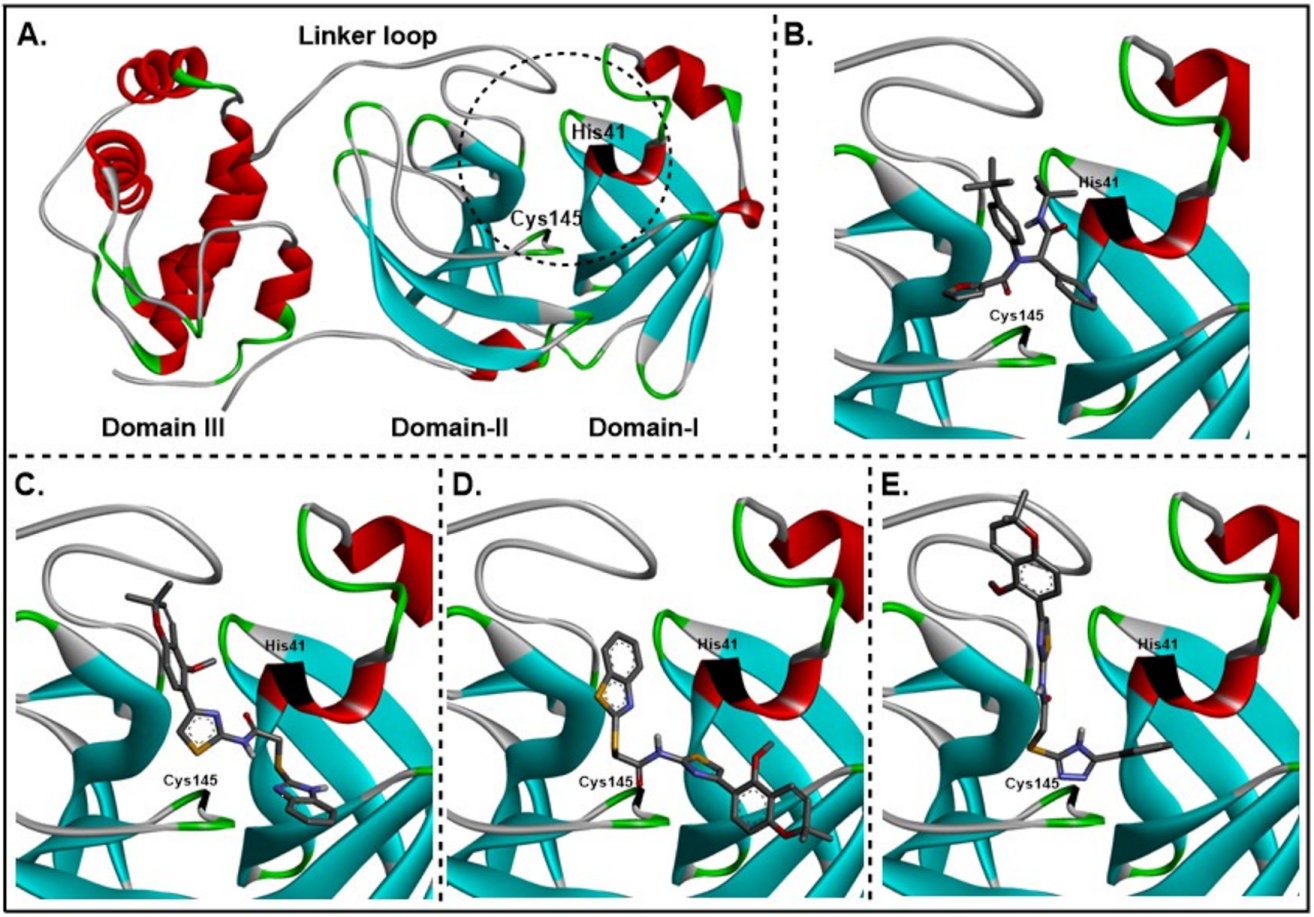
Orientation of ML188 and the chromene-thiazole derivatives **5–7** in the SARS-CoV-2 M^pro^ (PDB ID: 6LU7) active site. (A) Enzyme with active site region circled. His41 and Cys145 residues are shown in black, **B-E:** Expanded views of compounds in active site. (B) ML188, (C) Compound **5**, (D) Compound **6**, (E) Compound **7**. The images were generated in Discovery Studio.

The validity of the ligand positions within the M^pro^ active site was verified by redocking the N3 inhibitor and superimposing the docked structure with the cocrystallized ligand (Figure 5). This process gave a docking affinity score of −7.1 kcal/mol, which is comparable to previous reports.^59,93^ The RMSD (root-mean-square deviation) value of 1.203 Å, is below the 2 Å threshold that is considered good agreement between the native ligand and redocked ligand.^59,93,94^

**Figure 5:**
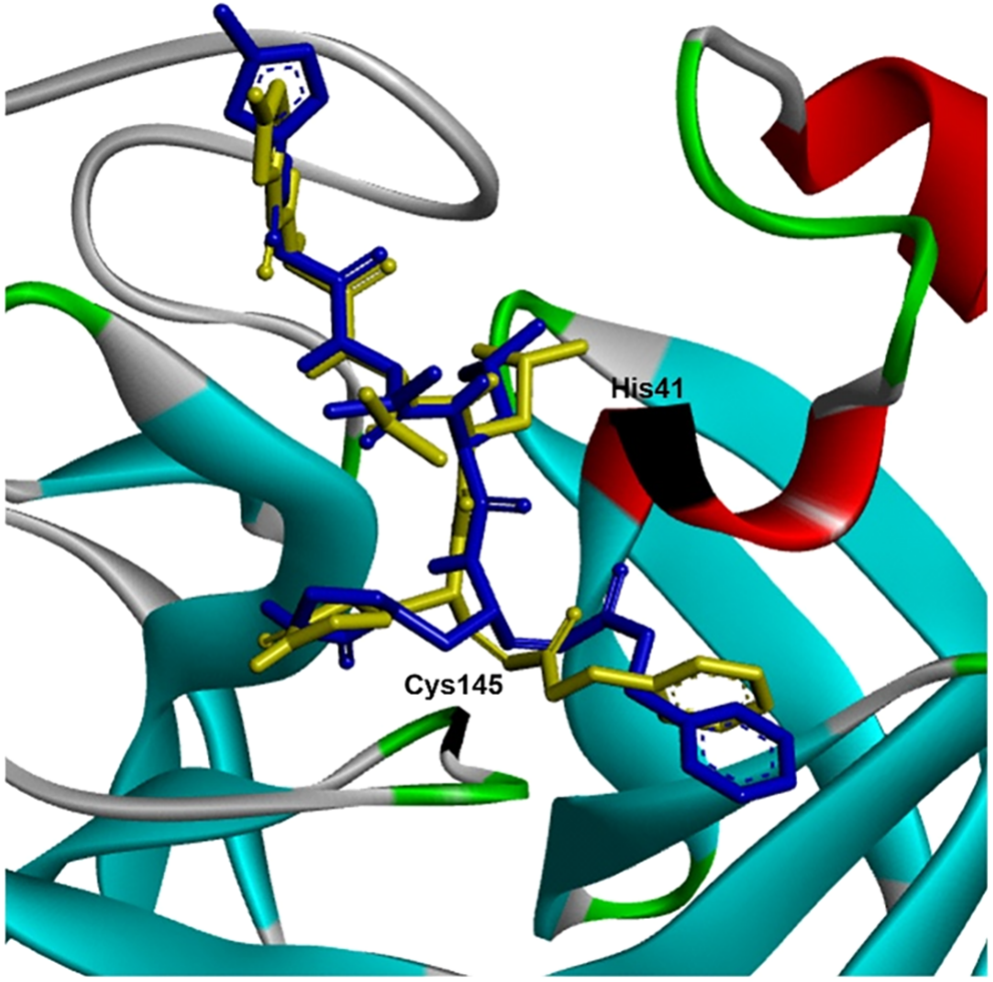
Superimposition of redocked N3-M^pro^ complex (blue) and cocrystallized complex (yellow) in the active site using Discovery Studio. (RMSD = 1.203 Å).

### 3.5 Molecular Dynamics Simulations Analysis

The rigid protein-flexible ligand model used for molecular docking provides a very good estimate of the interactions of the ligands with the protein. However, more accurate data is obtained by molecular dynamics using a flexible protein-flexible ligand model, which allows for an analysis of the conformational adjustment of the protein–ligand complex. Molecular dynamics has an added advantage because the simulations are carried out in solvent, thereby mimicking the biological environment. Thus, molecular dynamics simulations were conducted on the chromene-thiazole derivatives (**5–7**) to assess the conformations derived from molecular docking with respect to the stability of the ligands in the M^pro^ binding site (Figure 6).

**Figure 6:**
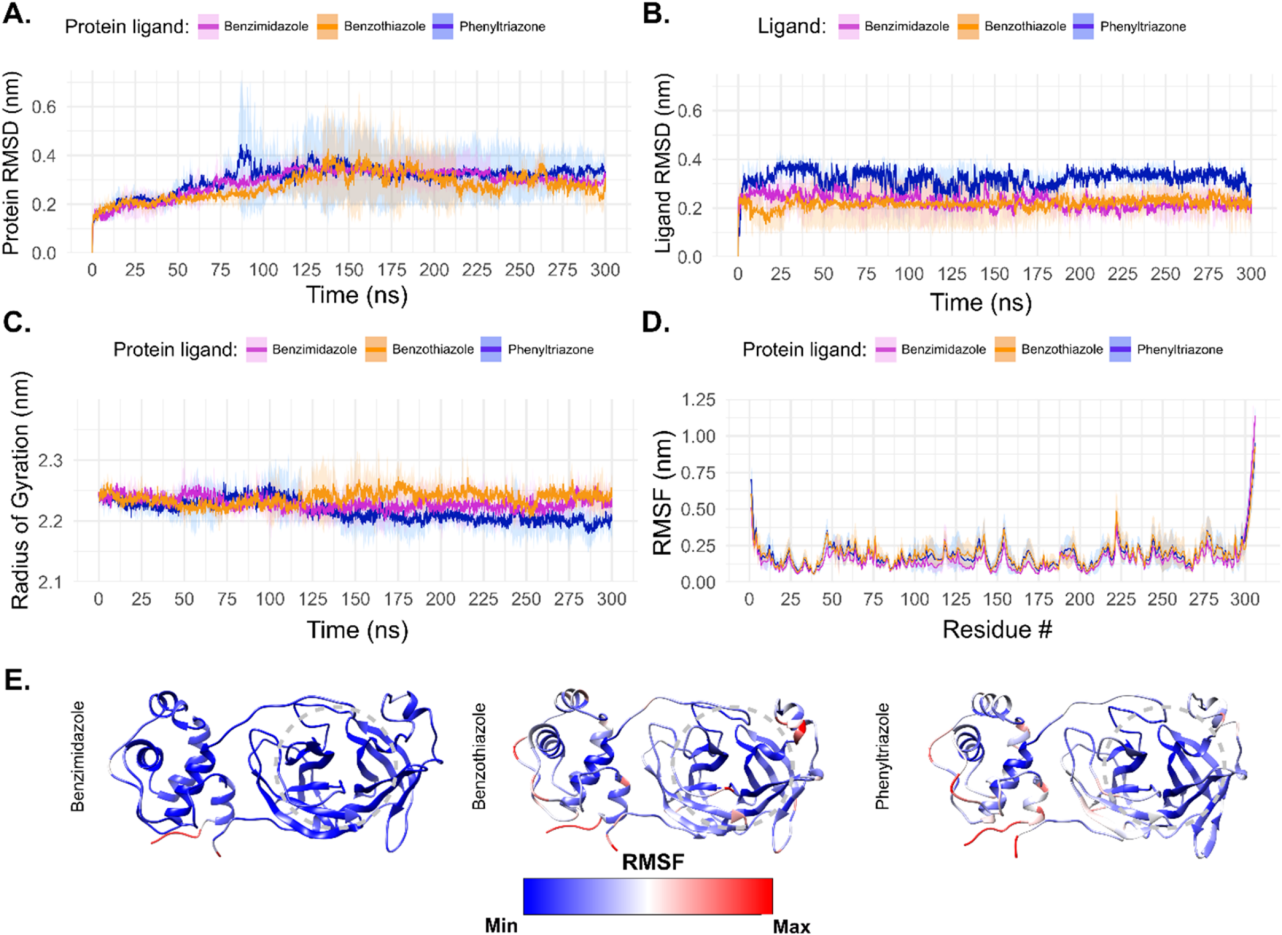
Comparative structural analysis of M^pro^ in complex with benzimidazole (**5**), benzothiazole (**6**), and phenyl-1,2,4-triazole (**7**) derivatives. (A) Root mean square deviation (RMSD) of M^pro^ backbone atoms over 300 ns MD simulations. Colored lines represent the average of three independent replicates, with shaded regions indicating the standard deviation among replicates. (B) RMSD of the ligands within the M^pro^ binding site, showing their mobility over time. Shaded areas replicate variability through standard deviation. (C) Temporal evolution of the protein radius of gyration (*R*_g_), indicating global compactness of M^pro^ throughout the simulations. (D) Root mean square fluctuation (RMSF) per residue of M^pro^ in complex with each ligand. Shaded areas represent standard deviations across replicates. (E) M^pro^ ribbon representation, colored on the basis of the RMSF value. The dashed circle highlights the binding site of the ligands. Individual RMSD and RMSF plots can be seen in the Figures S21–23.

Figure 6A illustrates the RMSD of the M^pro^ protein backbone atoms during the MD simulations involving the three ligands. The pink line represents the benzimidazole complex, which exhibited stable fluctuations after roughly 125 ns, with an average RMSD of approximately 0.35 nm. The narrow shaded region around the curve signifies minimal standard deviation among replicates, indicating a high degree of consistency across independent simulations. The benzothiazole complex (orange line) exhibited strong consistency among replicates up to approximately 125 ns, followed by a phase of significant stability from 125 to 200 ns. The replicates stabilized beyond this interval, exhibiting an RMSD near 0.30 nm, which signifies convergence of the protein–ligand system.

The phenyl-1,2,4-triazole complex (blue line) demonstrated the greatest variability among the three ligands. The shaded area significantly increased between 75 and 100 ns, indicating considerable variability among replicates. The variability, while reduced after 100 ns, continued to exhibit some fluctuations during the simulation. At the conclusion of the trajectory, the mean RMSD reached approximately 0.30 nm, comparable to the other ligands, yet with increased inter-replicate variability. Overall, the observed average for all replicates is very small, and it is in accordance with previous MD simulations using the M^pro^ in complex with other ligands.^95,96^ The stability pattern of the three derivatives mimics the trend observed from molecular docking.

Figure 6B illustrates the RMSD profiles of the three ligands located within the M^pro^ binding site. The benzimidazole ligand (pink line) demonstrated a high level of agreement among replicates, as evidenced by the minimal shaded area indicating the standard deviation. Following roughly 100 ns of simulation, the RMSD stabilized at approximately 0.20 nm, indicating minimal mobility of the ligand within the active site. The benzothiazole ligand (orange line) exhibited a relatively stable mean RMSD throughout the simulation, reaching stabilization around 50 ns. The shaded area exhibited significant variability among replicates. One replicate exhibited a notably low RMSD, with the shaded area nearing the 0.10 nm line, suggesting that this trajectory maintained high stability within the binding site. The mean RMSD for the benzothiazole ligand was approximately 0.20 nm. The phenyl-1,2,4-triazole ligand (blue line) exhibited the greatest degree of movement within the binding site, with RMSD values nearing 0.30 nm and displaying significant fluctuations throughout the trajectory. Despite these fluctuations, the shaded area was comparatively subtle when contrasted with benzothiazole ligand, suggesting a higher degree of inter-replicate agreement. A more significant stabilization of the phenyl-1,2,4-triazole ligand was observed around 200 ns of simulation. The results indicate that benzimidazole ligand exhibited the most consistent behavior across replicates, whereas the benzothiazole ligand demonstrated the highest overall stability within the binding site.

The analysis of interaction types over time indicates that the benzothiazole ligand establishes the highest number of contacts with the protein, especially toward the conclusion of the simulation, suggesting a progressive stabilization of the complex (Figure S25). His41 is involved in hydrogen bonding and, more significantly, π-stacking interaction with the ligand, particularly in replicate 1 and replicate 3. The benzimidazole complex exhibits an intermediate and more variable number of interactions among replicates. For example, His41 demonstrates hydrogen bonding and π- stacking solely in replicate 1, while these interactions are significantly less pronounced in replicates 2 and 3, with no observable increase in contacts noted toward the conclusion of the simulations (Figure S24).

Phenyl-1,2,4-triazole ligand exhibits the least number of interactions with the protein overall. In replicates 1 and 3, significant blank regions at the beginning and end of the interaction plot reflect a lack of contacts during those intervals. Replicate 1 successfully established interactions, notably with residues Pro184, Phe185, and Val186. Replicate 2 demonstrated an exception, exhibiting a greater number of interactions, including increased contacts with Cys44 and π-stacking with His41, similar to the observations made for benzimidazole, although in smaller amounts (Figure S26). Figure 6C presents the temporal evolution of the radius of gyration (Rg) of M^pro^ in complex with the three ligands. The Rg parameter indicates the overall compactness of the protein throughout the simulation; an increase in Rg indicates unfolding, while a decrease implies a more compact, folded state. The complexes with benzothiazole and benzimidazole exhibited nearly constant Rg values, indicating that the global compactness of M^pro^ remained largely unchanged in the presence of these ligands, with no evidence of unfolding or increased packing. The phenyl-1,2,4-triazole complex demonstrated a slight decrease in Rg over time, indicating a modest enhancement in protein compactness.

Figure 6D illustrates the residue root-mean-square fluctuation (RMSF) profiles of M^pro^ for the three ligand-bound systems. The most flexible regions are the C- and N-terminals, which was to be expected since there are fewer structural interactions in these regions and they are highly exposed to the solvent. The observed fluctuations exhibit significant concordance across complexes and between replicates, as evidenced by the subtle shaded areas denoting standard deviations. The benzimidazole complex exhibits consistently lower RMSF values throughout the trajectory in comparison to benzothiazole and phenyl-1,2,4-triazole, indicating a more stable protein–ligand interaction. For the benzothiazole and phenyl-1,2,4-triazole complexes, minor alternations are observed in which one complex exhibits slightly lower or higher fluctuations relative to the other at different regions of the protein. Figure 6E shows the RMSF fluctuation structurally. The M^pro^-benzimidazole complex shows a very stable protein, as most of the protein is colored blue, except for the N- and C-terminus. The other two complexes show a similar behavior, with some parts showing intermediate RMSF fluctuation (white color), especially the loop regions. Other studies have also shown this behavior for the M^pro^ protein, where the loop and N- and C-terminals show high fluctuation.^28^ All three replicates present the binding site (highlighted by the dashed circle) in a very stable manner, including the loop around it. This region is known for high fluctuation and previous analyses have suggested that an inhibitor in the binding site may stabilize the surrounding loop, as observed here.^97^ These results suggest that the MD simulations were successful for their purpose.

According to the QM/MM study conducted using the QM-MM/GBSA approach, all replicas and the three complexes had average energies below zero, indicating the creation of an energetically favorable complex (Table 1). The average binding free energies (standard deviation) calculated for the M^pro^–benzimidazole, M^pro^–benzothiazole, and M^pro^–phenyl-1,2,4-triazole complexes were −17.71 ± 4.25, −17.69 ± 5.92, and −7.33 ± 5.95 kcal/mol, respectively. The only complex with values greater than 0 as the maximum value was the M^pro^-phenyl-1,2,4-triazole complex. The conformation of replicate 1 has the lowest energy in the benzimidazole complex and is −32.22 kcal/mol (frame 2513). The complex containing benzothiazole was derived from the simulation of replicate 2 and has an energy value of −34.4 kcal/mol (frame 2915), while the complex with phenyl-1,2,4-triazole showed the lowest energy value of replicate 2 at −24.90 kcal/mol (frame 2913). In this context, the M^pro^ showed a higher affinity for benzothiazole, followed by benzimidazole and phenyl-1,2,4-triazole as the lower affinity.

**Table 1:** Summary of the QM/MM Calculations Results from the Last 50 ns of Each Replica of the MD Simulation

**ligand**	**replica**	**min**	**Q1** ^a^	**median**	**mean**	**Q3** ^b^	**max**	**SD** ^c^
**5** (benzimidazole)	1	–**32.22**	–21.87	–19.42	–19.26	–16.5	–7.07	3.98
	2	–29.4	–19.4	–16.6	–16.6	–13.8	–4.2	3.92
	3	–26.79	–20.32	–17.35	–17.25	–14.6	–3.63	4.39
**6** (benzothiazole)	1	–26.15	–18.49	–14.84	–15.02	–11.20	–3.81	4.61
	2	–**34.4**	–26.10	–23.4	–23.7	–21.2	–11.1	3.68
	3	–27.94	–16.90	–14.15	–14.38	–11.66	–4.19	4.05
**7** (phenyl-1,2,4-triazole)	1	–17.32	–18.49	–7.11	–3.53	–0.71	10.60	4.61
	2	–**24.90**	–26.10	–14.68	–11.68	–8.47	8.90	4.75
	3	–20.69	–16.90	–10.84	–6.89	–2.73	8.33	5.83

a First quartile.

b Third quartile.

c Standard deviation.

Figure 7 shows the average of the QM/MM calculations of the three replicates of the three complexes. It can be seen that most of the averages are below 0 kcal/mol, and the benzimidazole and benzothiazole have shown similar averages in the last 50 ns of the simulation. To perform a more precise affinity calculation, we chose the frame with the lowest energy of each complex and performed the QM calculation using the DFT formalism together with the MFCC approach.

**Figure 7:**
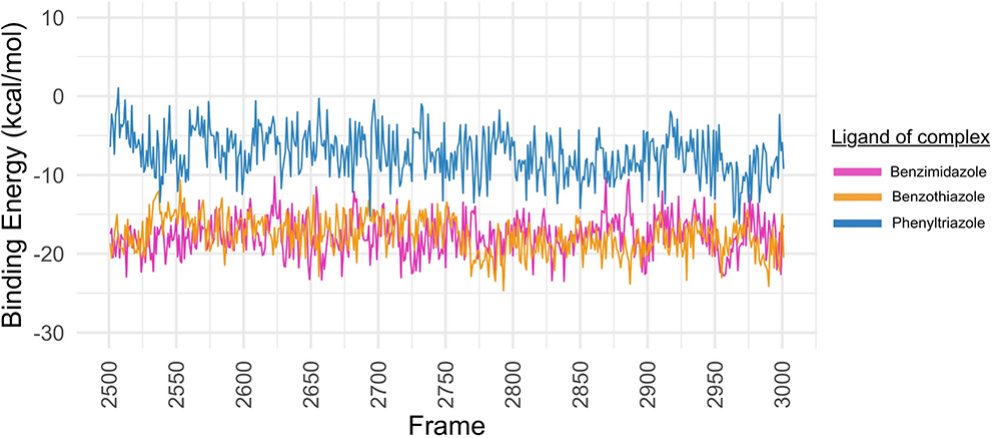
Average QM/MM analysis of the last 500 conformational modes (50 ns of MD simulation) of the M^pro^-benzimidazole (pink line), M^pro^-benzothiazole (orange line) and M^pro^-phenyl-1,2,4-triazole (blue line). The QM/MM plot for individual replicas can be seen in Figures S27–29.

### 3.6 Quantum-Mechanical Calculations

QM calculations are the most accurate method for calculating *in silico* protein–ligand binding affinity. However, the computational cost for large systems, such as proteins, is high and the time required is not worth it. The MFCC approach is a reliable way to enable QM for such systems.

Figure 8 shows the results of de QM employing MFCC to reduce the computational load. Convergence for both dielectric constants, ε = 10 and 40, were obtained for benzimidazole at a radius of *r* = 7.0 Å (ε = 10: −65.09 kcal/mol; ε = 40: −63.43 kcal/mol), according to our convergence criteria shown in Figure 8A–C. This corresponds to a deviation of less than 10% from their *r* = 6.5 Å values (Figure 8A). The radius of convergence for benzothiazole was 6.0 Å (ε = 10: −63.70 kcal/mol; ε = 40: −62.23 kcal/mol) (Figure 8B), while the radius of convergence for phenyl-1,2,4-triazole was 7 Å for both constants (ε = 10 and 40) at −69.40 kcal/mol and −66.81 kcal/mol, respectively (Figure 8C). This indicates that the most important amino acids for binding affinity were considered in the calculation. In all convergence analyzes, ε = 10 had a lower energy value than ε = 40, indicating that our calculations were performed successfully, as shown in previous studies.

**Figure 8:**
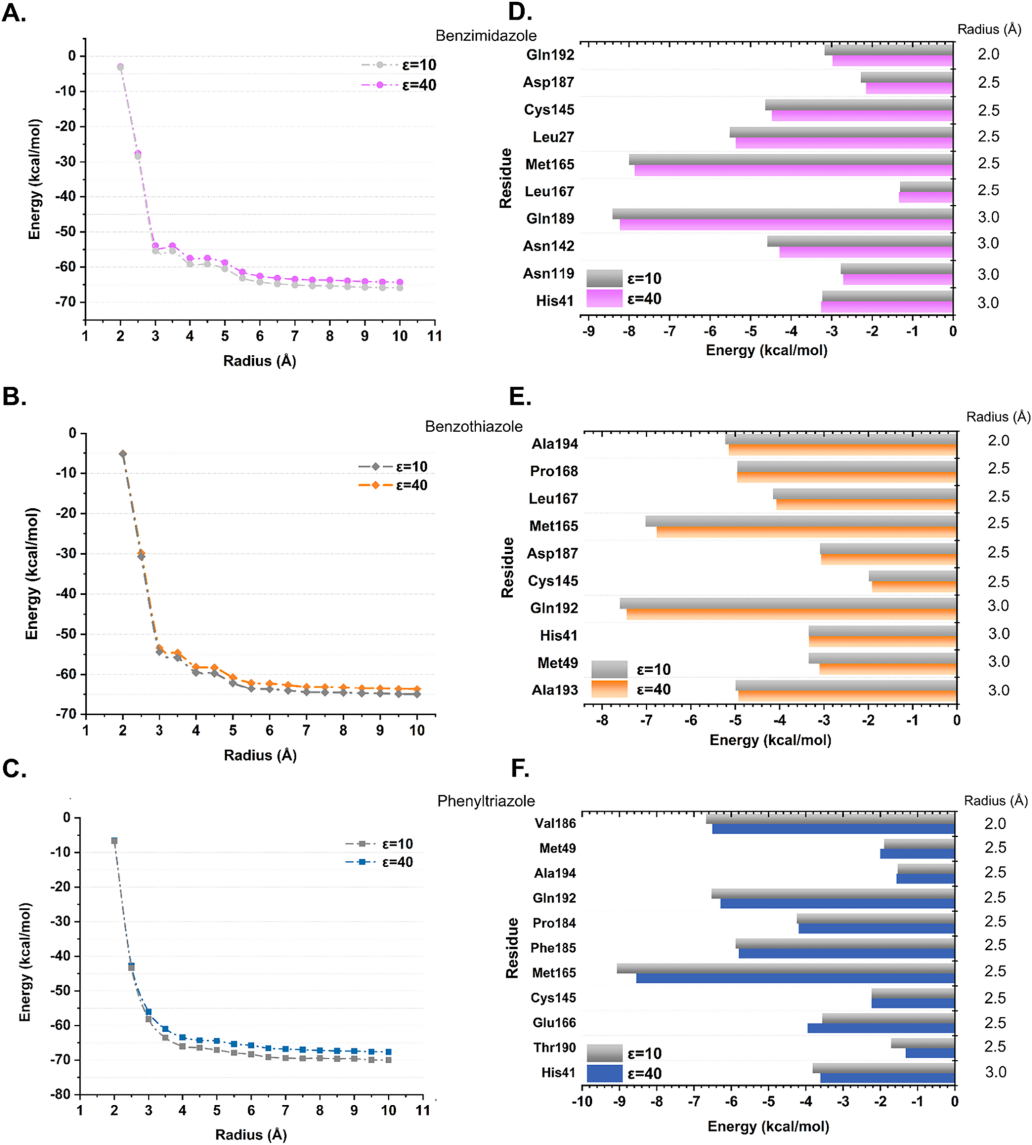
Analysis of energy from QM calculations. (A–C) Convergence plot showing the calculated energy (in kcal/mol) as a function of the distance of the ligand (radius in Å). (D–F) Bar chart of the energy in kcal/mol of the residues that contribute most to the binding affinity. The color of the energy for the dielectric constant ε = 10 is in shades of gray, and for ε = 40 it is pink, orange and blue for benzimidazole (**5**), benzothiazole (**6**) and phenyl-1,2,4-triazole (**7**), respectively.

As previously mentioned, we adopted two distinct dielectric values to represent the solvation environment and its effects in both ligands. However, from now on, we will limit the discussion of our results to the dielectric value ε = 40, a better theoretical value for proposed *in vivo* simulations.^76^ Taking into account *r* = 10 Å, we have these values of binding affinity in descending order of affinity: phenyl-1,2,4-triazole (−67.63 kcal/mol), benzimidazole (−64.28 kcal/mol) and benzothiazole (−63.69 kcal/mol), which is consistent with the trend from molecular docking. Although these results are not consistent with the QM/MM calculations, previous analyses have shown that DFT agrees better with the experimental data and the QM/MM calculations are useful to select the complex for QM analysis.^98,99^

Figure 8D–F shows the bar chart of interaction energies of M^pro^ amino acid residues with the three derivatives. For the benzimidazole derivative (**5**), the descending order of affinity of the residues is as follows: Gln189 (−8.23 kcal/mol) > Met165 (−7.86 kcal/mol) > Leu27 (−5.27 kcal/mol) > Cys145 (−4.48 kcal/mol) > Asn142 (−4.29 kcal/mol) > His41 (−3.26 kcal/mol) > Gln192 (−2.98 kcal/mol) > Asn119 (−2.72 kcal/mol) > Asp187 (−2.15 kcal/mol) > Leu167 (−1.34 kcal/mol). For the interaction between M^pro^ and the benzothiazole derivative (**6**), the most important residues are Gln192 (−7.45 kcal/mol), Met165 (−6.77 kcal/mol), Ala194 (−5.15 kcal/mol), Pro 168 (−4.96 kcal/mol), Ala193 (−4.93 kcal/mol), Leu167 (−4.07 kcal/mol), His41 (−3.34 kcal/mol), Met49 (−3.1 kcal/mol), Asp187 (−3.07 kcal/mol), and Cys145 (−1.92 kcal/mol). Finally, the affinities of the amino acid residues for the phenyl-1,2,4-triazole derivative (**7**) are Met165 (−8.55 kcal/mol), Val186 (−6.51 kcal/mol), Gln192 (−6.29 kcal/mol), Phe185 (−5.80 kcal/mol), Pro184 (−4.19 kcal/mol), Glu166 (−3.95 kcal/mol), His41 (−3.61 kcal/mol), Cys145 (−2.24 kcal/mol), Met49 (−2.0 kcal/mol), Ala194 (−1.57 kcal/mol), and Thr190 (−1.32 kcal/mol).

BIOVIA Discovery Studio Client was used to perform the interaction analyses of the derivatives with the higher affinity M^pro^ amino acid residues, together with His41 and Cys145, which make up the catalytic dyad. As displayed in Figure 9A, the benzimidazole derivative (**5**) forms a pi-pi stacked interaction with His41 and a pi-alkyl and hydrogen bond with Cys145. The Met165 residue, which had the second highest affinity for the benzimidazole derivative, showed three interactions: two pi-alkyl and one alkyl–alkyl. Another important residue for the interaction with this ligand is Leu27, which forms a pi-alkyl interaction with the molecule. Asn142 and Gln192 form hydrogen bonds and Gln189, which displays the most stable interaction, has its affinity essentially through van der Waals forces.

**Figure 9:**
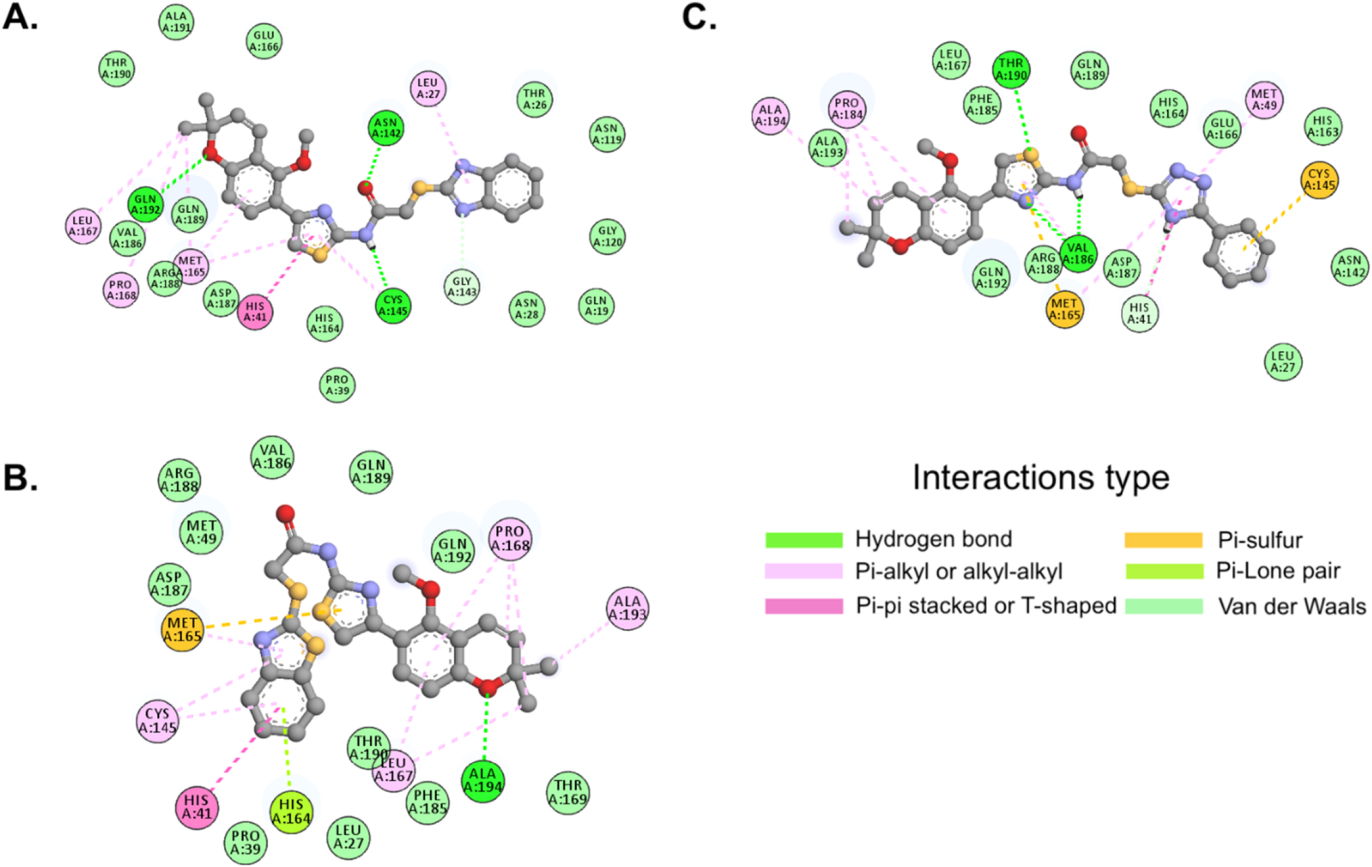
2D interaction maps for the M^pro^ in complex with chromene-thiazole derivatives. (A) benzimidazole (**5**), (B) Benzothiazole (**6**), (C) Phenyl-1,2,4-triazole (**7**).

The major amino acid residues interacting with the benzothiazole derivative (**6**) are shown in Figure 9B. The benzothiazole derivative displays a pi-pi stacked interaction with His41, as was also observed with benzimidazole. The Cys145 residue forms two pi-alkyl interactions. The Met165 residue, which also has the second highest affinity for the ligand, forms one pi-sulfur and one pi-alkyl interaction. Interaction with Gln192, the residue with the highest affinity for the benzothiazole, is essentially driven by van der Waals forces. Ala193 and Ala194 also showed good affinity for the benzothiazole derivative, forming alkyl–alkyl and hydrogen bonding interactions, respectively.

The interactions of key amino acid residues with the phenyl-1,2,4-triazole derivative (**7**) are presented in Figure 9C. The phenyl-1,2,4-triazole derivative forms pi-pi T-shaped and pi-donor hydrogen bond interactions with His41 and a pi-sulfur interaction with Cys145. Upon association with the phenyl-1,2,4-triazole derivative, Met165, the amino acid residue with the highest affinity, forms pi-sulfur and pi-alkyl interactions. Val186, another high affinity residue, forms two hydrogen bonds. Phe185 and Gln192 also showed good affinity, which is mainly driven by van der Waals forces, while the lower affinity Pro184 residue forms three interactions: two pi-alkyl and one alkyl–alkyl interaction.

Based on Figures 8D–F and 9A–C, the amino acids that were related in all three complexes were the catalytic dyad, His41 and Cys145, as well as Met165 and Gln192. Interaction types and distances for the three chromene-thiazole ligands with these amino acid residues are presented in Table 2. Interaction distances range from 4.04 to 7.65 Å. In addition to the catalytic dyad, the amino acid Met165 has been described as an important hotspot for the binding of inhibitors.^100,101^ It is important to note that for all three complexes, Met165 contributed significantly to the binding affinity (Figure 8D–F). Moreover, the stability of the interaction for this residue follows the trend observed for the docking affinity of the ligands: phenyl-1,2,4-triazole (**7**) > benzimidazole (**5**) > benzothiazole (**6**). Furthermore, it has also been reported that Gln192 plays a crucial role in the formation of the active site.^102^ The benzothiazole derivative (**6**) forms the most stable interaction with Gln192, and significant interaction is also observed for the phenyl-1,2,4-triazole derivative (**7**)

**Table 2:** Docking Interactions of Compounds **5-7** with His41, Cys145, Met165 and Gln192 in the Active Site of SARS-CoV-2 M^pro^ (PDB ID: 6LU7)

**ligand**	**amino acid**	**interaction**	**distance (Å)**
**5** (benzimidazole)	His41	pi-pi stacked	4.91
	Cys145	hydrogen bond	4.18
		alkyl/pi-alkyl	7.65
	Met165	alkyl/pi-alkyl	4.47/4.86/5.03
	Gln192	hydrogen bond	4.64
**6** (benzothiazole)	His41	pi-pi stacked	5.12
	Cys145	alkyl/pi-alkyl	5.65/7.38
	Met165	pi-sulfur	6.37
		alkyl/pi-alkyl	4.52
	Gln192	van der Waals	NA
**7** (phenyl-1,2,4-triazole)	His41	pi-pi T-shaped	6.11
		pi-donor hydrogen bond	4.04
	Cys145	pi-sulfur	5.97
	Met165	pi-sulfur	7.43
		alkyl/pi-alkyl	4.10
	Gln192	van der Waals	NA

As noted previously, the phenyl-1,2,4-triazole derivative (**7**) exhibited the most favorable docking score (−8.4 kcal/mol) and prompted a decrease in M^pro^ radius of gyration during molecular dynamics simulations, aligning with ligand-induced compaction of the binding pocket. Two complementing causes are likely responsible for this behavior. The docking arrangement positions the phenyl-1,2,4-triazole unit near the catalytic dyad (His41–Cys145), optimizing van der Waals contacts and π–stacking interactions with active-site residues (Figure 9C). The attached phenyl ring on the 1,2,4-triazole scaffold enables greater penetration of the triazole moiety into the pocket, relative to the benzimidazole and benzothiazole units, while offering conformational flexibility that enhances the ligand’s optimal arrangement within the site (Figure 10). This configuration amplifies the buried surface area, elucidating the noted drop in Rg and the enhanced docking and QM/MM stability of compound **7** compared to the benzimidazole and benzothiazole equivalents. Hence, the phenyl-1,2,4-triazole derivative has structural properties that enhance the buried surface area and promotes stronger short-range interactions between protein and ligand in the pocket. The greater docking affinity could also be attributed to electronic factors. Of the three derivatives, the phenyl-1,2,4-triazole derivative had the lowest E_gap_, and also displayed the best electron-accepting ability. These parameters are indicative of potentially stronger interactions with electron-rich sites in the M^pro^ active site.

**Figure 10:**
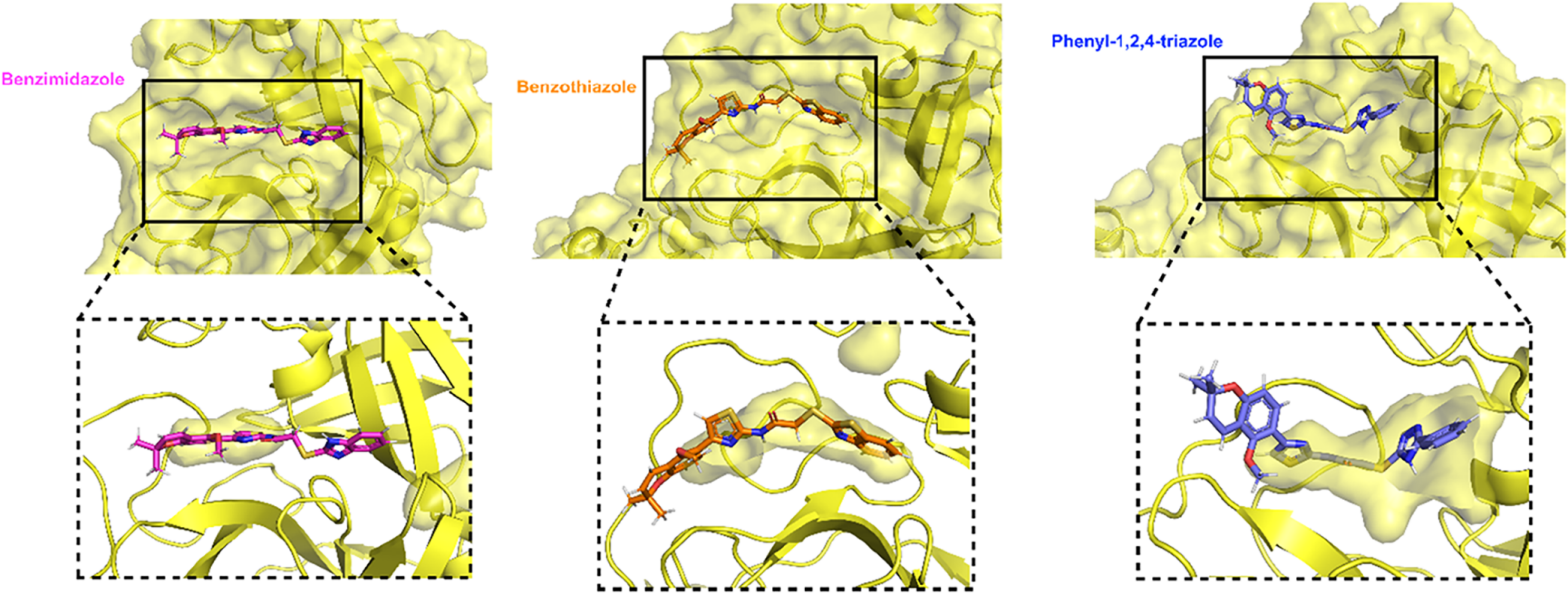
Binding poses of the three chromene-based derivatives within the M^pro^ active site. The M^pro^ protein is represented as a yellow surface and ribbon. Ligands are shown as sticks: benzimidazole derivative (pink), benzothiazole derivative (orange), and phenyl-1,2,4-triazole derivative (blue). The lower panels show enlarged views of the binding pocket (highlighted by dashed boxes) illustrating the deeper insertion of the phenyl-1,2,4-triazole derivative into the active site compared with the other ligands. The snapshots were retrieved from the same MD/replicate of the QM analysis.

## 4 Conclusions

Three chromene-thiazole derivatives, incorporating benzimidazole, benzothiazole, and phenyl-1,2,4-triazole moieties, were synthesized and characterized by spectroscopic studies. DFT analyses were performed to determine the chemical reactivity, with the phenyl-1,2,4-triazole derivative predicted to have the greatest reactivity and electron-accepting ability based on FMO band gap energy. Furthermore, all of the compounds showed potential for binding to biological targets based on the MEP analyses. Molecular docking analyses of the derivatives with SARS-CoV-2 M^pro^ indicated good docking affinity, with docking scores ranging from −7.5 to −8.4 kcal/mol, which are similar to or better than those obtained for ML188 (−7.5 kcal/mol). The interactions of the derivatives with M^pro^ were further explored by molecular dynamics simulations and QM/MM analyses. These studies supported the trend observed from molecular docking studies (phenyl-1,2,4-triazole > benzimidazole > benzothiazole). The higher affinity for the phenyl-1,2,4-triazole derivative is predicted by FMO analysis. The benzothiazole derivative is predicted to have higher affinity than benzimidazole derivative based on the reactivity parameters. However, the opposite was observed. Nevertheless, the molecular dynamics simulations and QM/MM data revealed stable interactions between key M^pro^ active site amino acid residues and the three chromene-thiazole derivatives. These include His41 and Cys145, which are involved in the M^pro^ catalytic mechanism, and Met165 and Gln192. These computational studies suggest that the chromene-thiazole scaffold incorporating benzimidazole, benzothiazole and phenyl-1,2,4-triazole moieties could be important in the development of new compounds targeting the SARS-CoV-2 M^pro^ enzyme. However, further *in vitro* and *in vivo* studies are needed to validate these findings.

## Supplementary Material



## Notes

Spectroscopic data (IR, ^1^H NMR, ^13^C NMR, HRMS) and images for molecular dynamics, QM calculations and molecular docking (PDF)
