# Supplementary material for: Chromene-Thiazole Derivatives as Potential SARS-CoV‑2 Mpro Inhibitors: Synthesis and Computational Studies
Source: ACS Omega. 2025 Dec 17;10(51):62757–74. doi: 10.1021/acsomega.5c07593 (PMC12756838; doi:10.1021/acsomega.5c07593)
Supplement: Supplementary file 1 [file ao5c07593_si_001.pdf]

SUPPORTING INFORMATION

Chromene-thiazole derivatives as potential SARS-CoV-2 M^{pro} inhibitors: Synthesis and computational studies

Lauren D. Stettler[a#], Vincent T. Kopysciansky[a#], Jenna E. Poor[a#], Gabriela de Lima Menezes[b#], Elton VanNoy[a], Guilherme Bastos Alves[c], Blake M. Shellenberger[a], Faith Garasich[a], Sylvia Stanell[a], Katyanna Sales Bezerra[c], Jonas Ivan Nobre Oliveira[c], Umberto Laino Fulco[b*], Geneive E. Henry[a*]

[a]*Department of Chemistry, Susquehanna University, 514 University Avenue, Selinsgrove, PA*

[b]*Bioinformatics Multidisciplinary Environment, Programa de Pós Graduação em Bioinformática, Universidade Federal do Rio Grande do Norte, Natal 59078-400, RN, Brazil*

[c]*Departamento de Biofísica e Farmacologia, Universidade Federal do Rio Grande do Norte, 59072-970, Natal-RN, Brazil*

* E-mail: henry@susqu.edu

*Email: umbertofulco@gmail.com

[#]L.D.S., V. T. K., J. E. P. and G. L. M. contributed equally to this work.

Table of Contents

Figures and Tables	Pages
Chromatography conditions for compounds **1-7**	S3
• Table S1	
HOMO-LUMO energies of chromene-thiazole derivatives (**5-7**)	S3
• Table S2	
Mass spectrometry data for compounds **3-7**	S4-S6
• Fig. S1-S5	
IR spectra for compounds **3-7**	S7-S9
• Fig. S6-S10	
NMR spectra for compounds **3-7**	S10-S14
• Fig. S11-S20	
Molecular Dynamics and QM Calculations of M^{pro} with compounds **5-7**	S15-S21
• Fig. S21-S29	

Table S1. Summary of chromatography conditions for compounds **1-7** using Teledyne RediSep gold columns.

**Cmpd.**	**Column size (g)/ Flow rate (mL/min)/ Fraction volume (mL)**	**Elution mode**	**Elution time (min)**
**1**	120(×2)/10-13/50	Normal phase (isocratic)<br>5% EtOAc-hexanes	25-160
**2**	120/15/50	Normal phase (isocratic)<br>5% EtOAc-hexanes	80-150
**3**	120/10/50	Normal phase (isocratic)<br>20% EtOAc-hexanes	60-195
**4**	40/6/30	Normal phase (isocratic)<br>15% EtOAc-hexanes	50-175
**5**	30/10/30	Reversed phase (gradient)<br>65% MeOH/water (0-35 min)<br>65% MeOH-70% MeOH/water (35-38 min)<br>70% MeOH/water (38-48 min)<br>70% MeOH-75% MeOH/water (48-50 min)<br>75% MeOH/water (50-95 min)	63-95
**6**	30/10/30	Reversed phase (gradient)<br>65% MeOH/water (0-19 min)<br>65% MeOH-75% MeOH/water (19-21 min)<br>75% MeOH/water (21-41 min)<br>75% MeOH-80% MeOH/water (41-43 min)<br>80% MeOH/water (43-100 min)	62-100
**7**	30/10/30	Reversed phase (gradient)<br>70% MeOH/water (1-25 min)<br>70% MeOH-75% MeOH/water (25-32 min)<br>75% MeOH/water (32-88 min)	55-80

Table S2. HOMO-LUMO energies of chromene-thiazole derivatives (**5-7**)

**Compound**	**E_{HOMO} (eV)**	**E_{LUMO} (eV)**	**E_{gap} (eV)**
**5**	-5.2407	-2.1799	3.0608
**6**	-5.2631	-2.4285	2.8346
**7**	-5.2865	-2.4998	2.7867

Mass spectrometry data

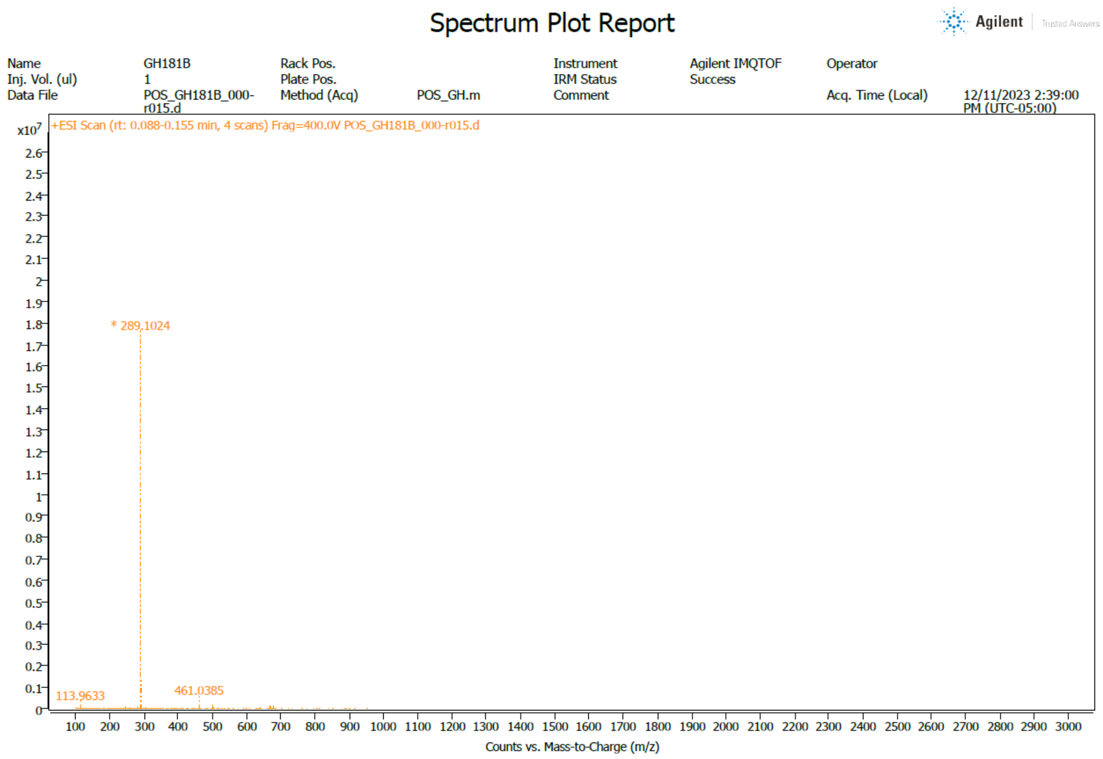

Figure S1. ESI-HRMS spectrum for compound **3**

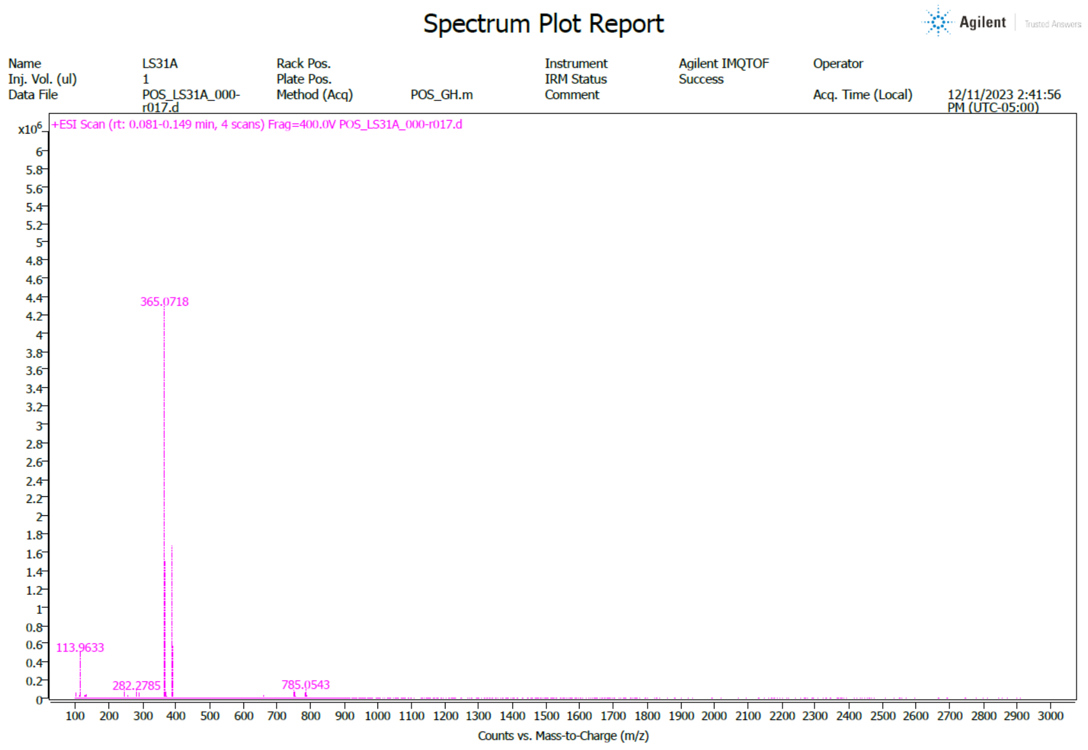

Figure S2. ESI-HRMS spectrum for compound **4**

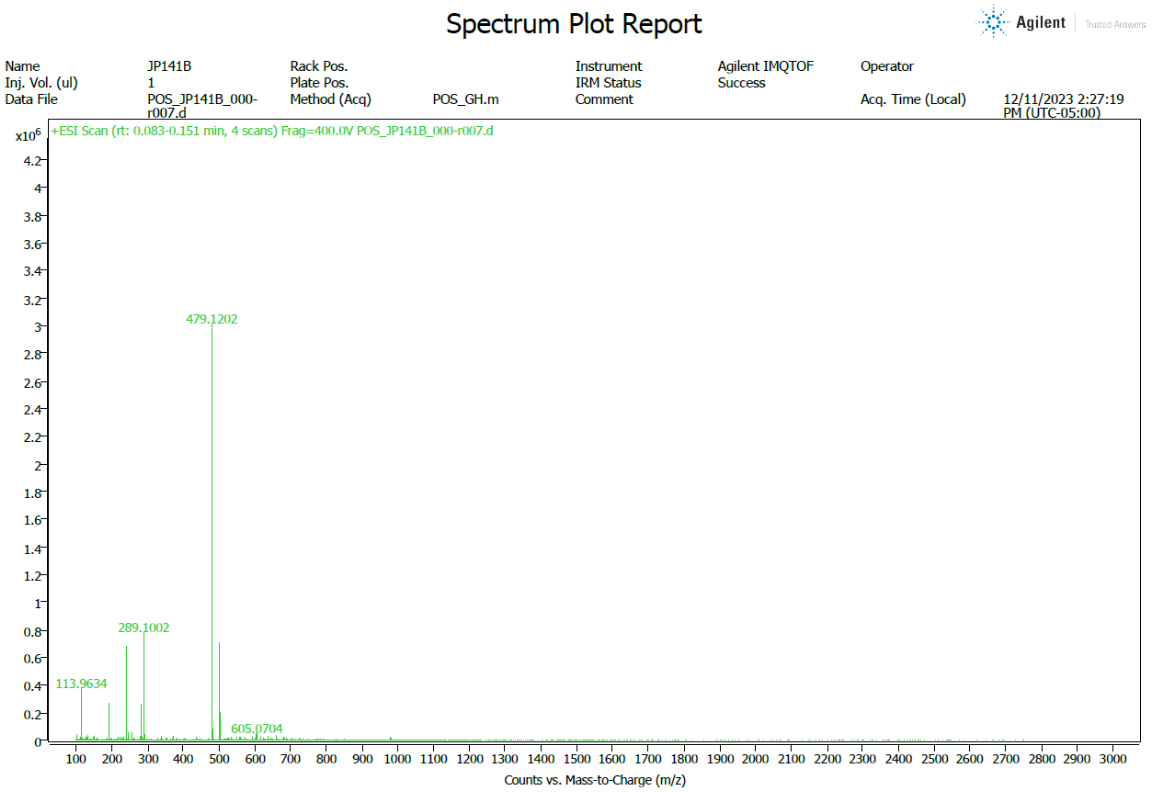

Figure S3. ESI-HRMS spectrum for compound **5**

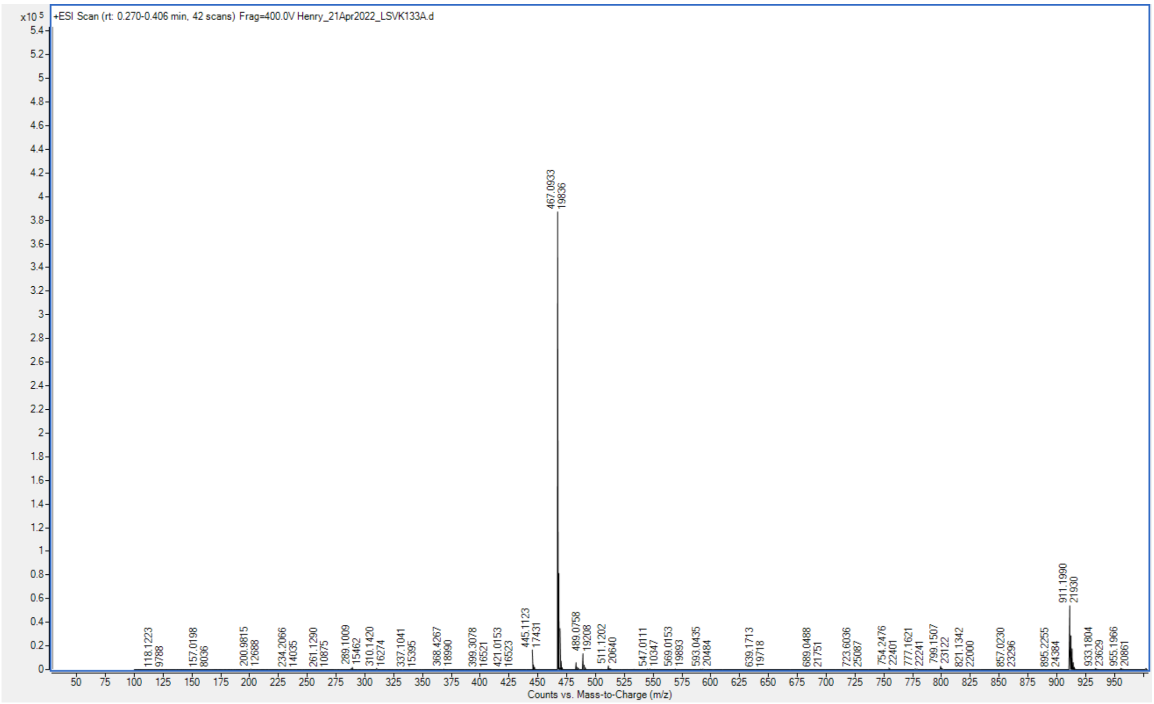

Figure S4. ESI-HRMS spectrum for compound **6**

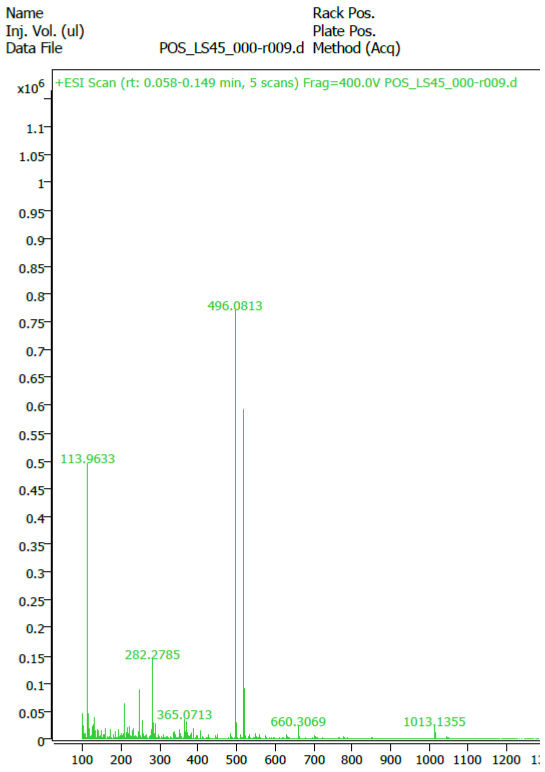

Figure S5. ESI-HRMS spectrum for compound **7**

IR spectra

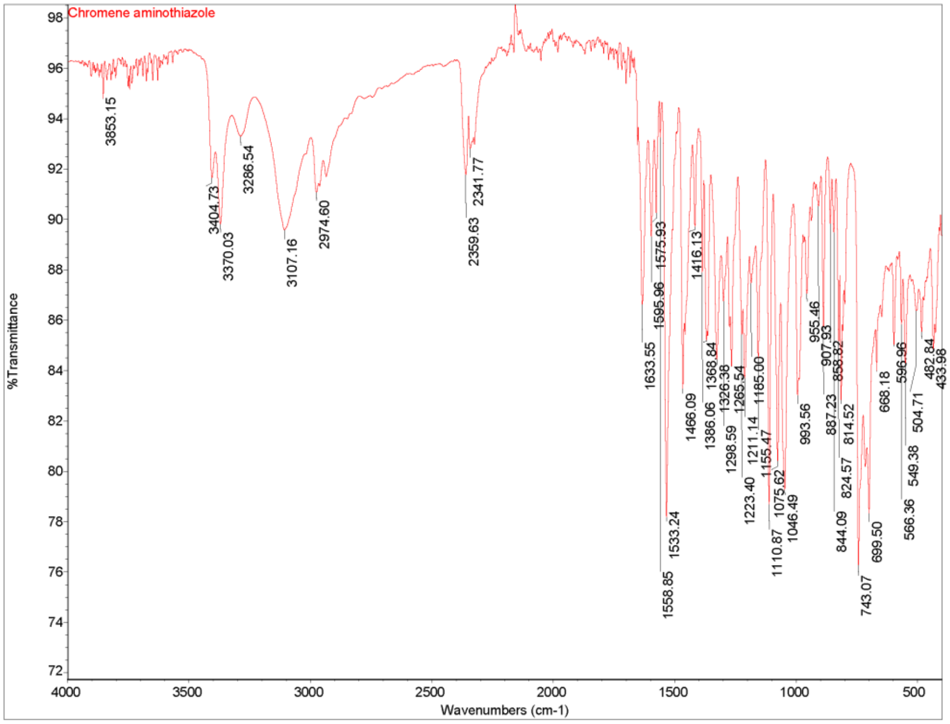

Figure S6. IR spectrum for compound **3**

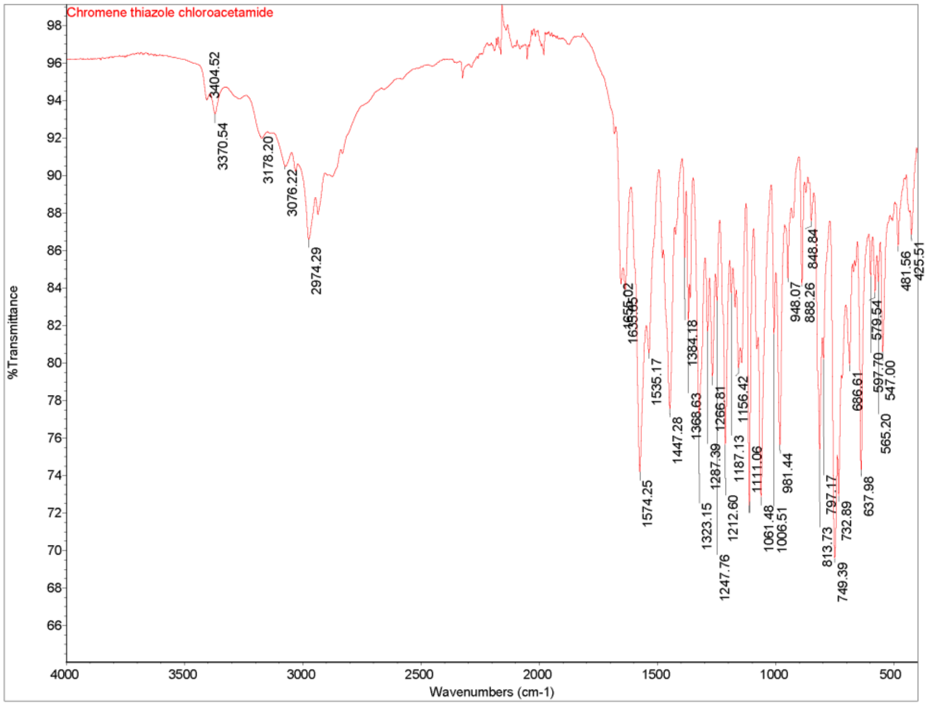

Figure S7. IR spectrum for compound **4**

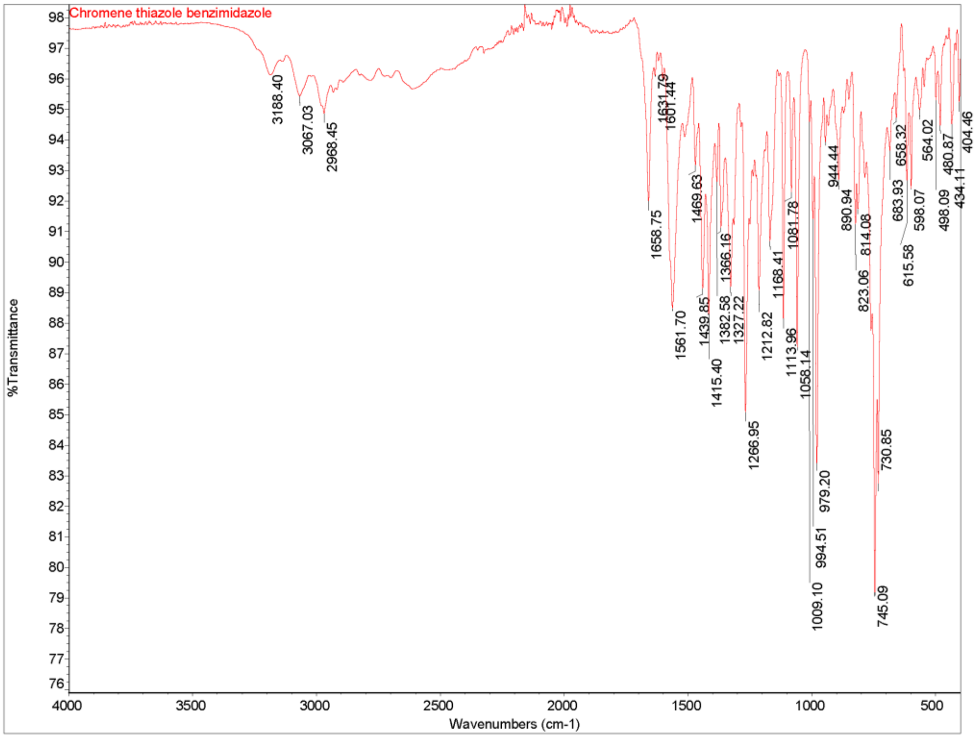

Figure S8. IR spectrum for compound **5**

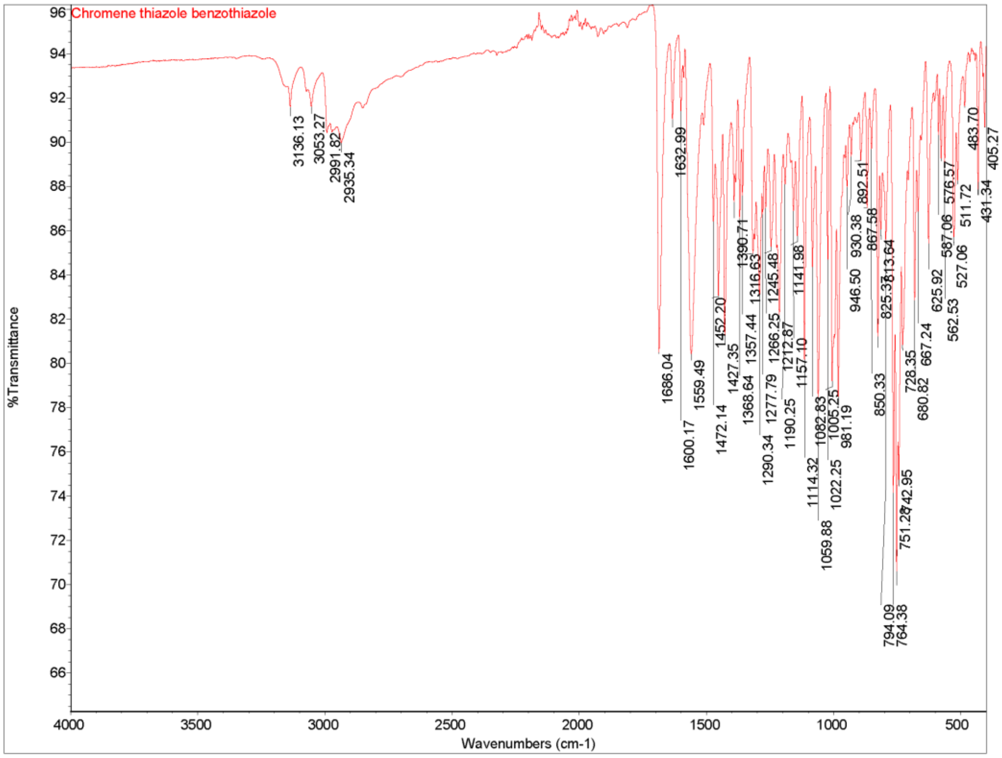

Figure S9. IR spectrum for compound **6**

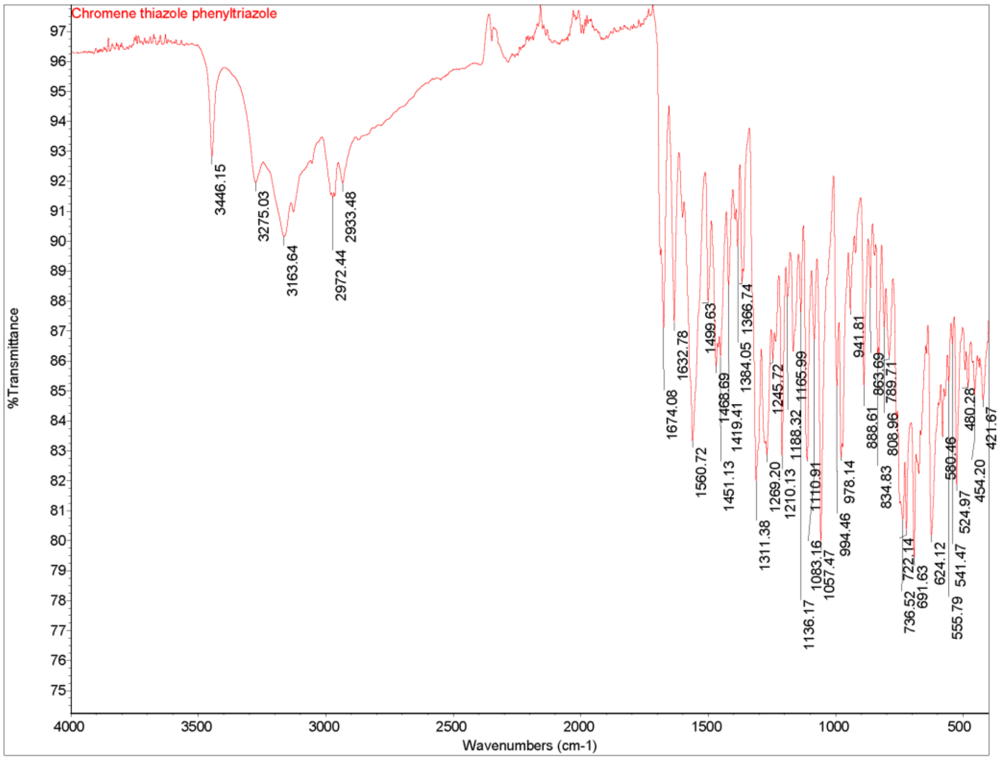

Figure S10. IR spectrum for compound **7**

NMR spectra

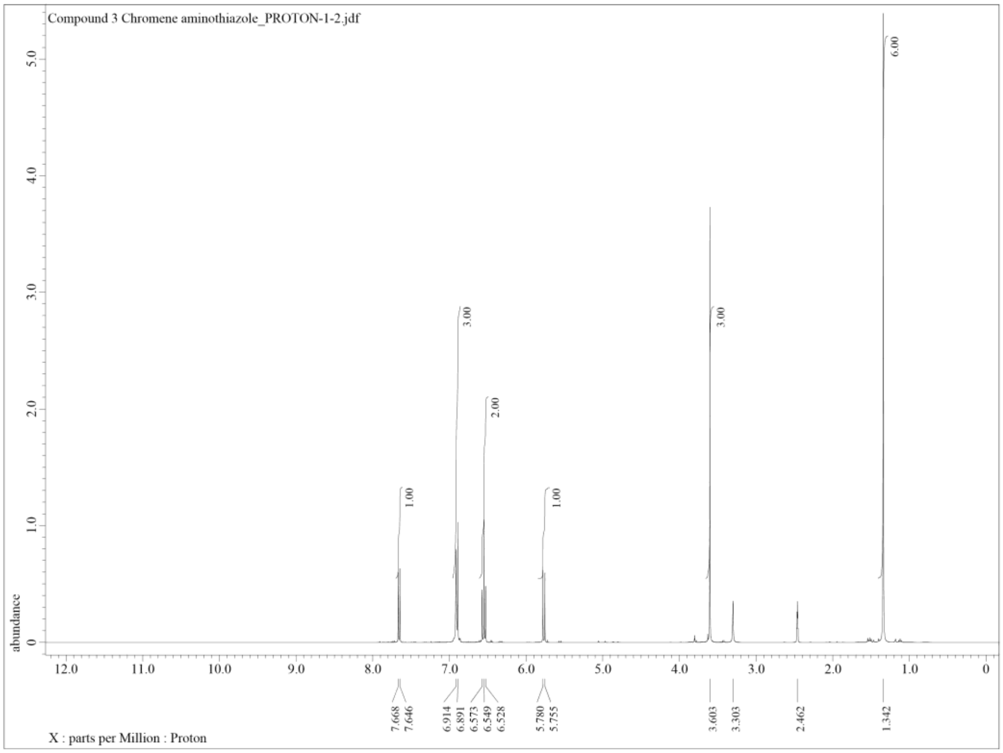

Figure S11. ^{1}H NMR spectrum for compound **3** in DMSO-d_6

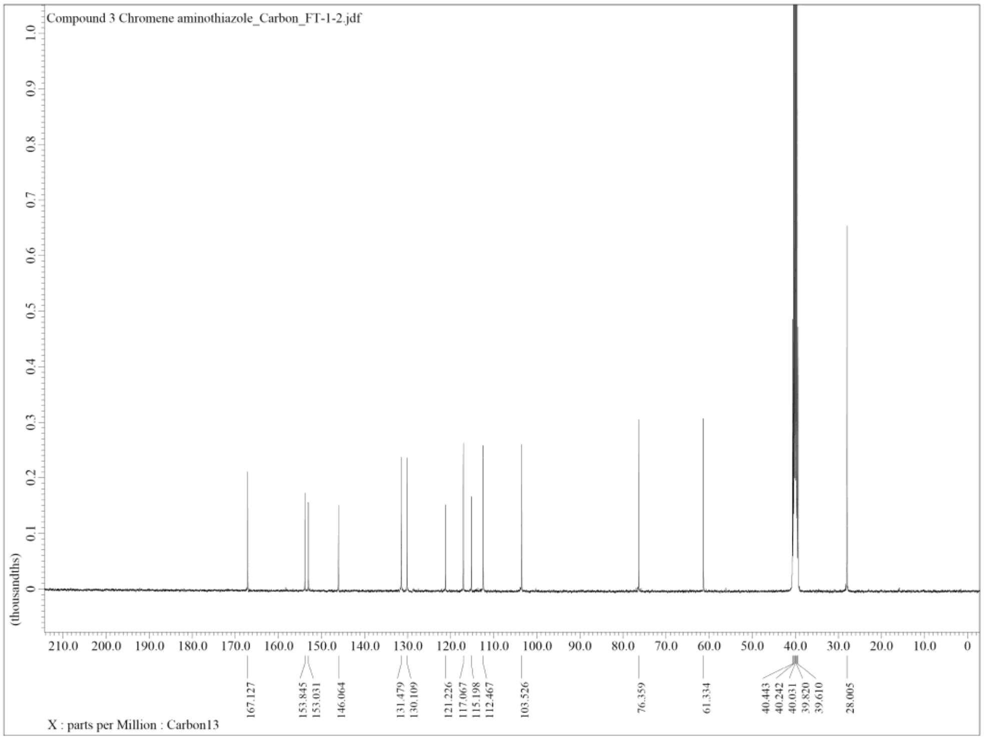

Figure S12. ^{13}C NMR spectrum for compound **3** in DMSO-d_6

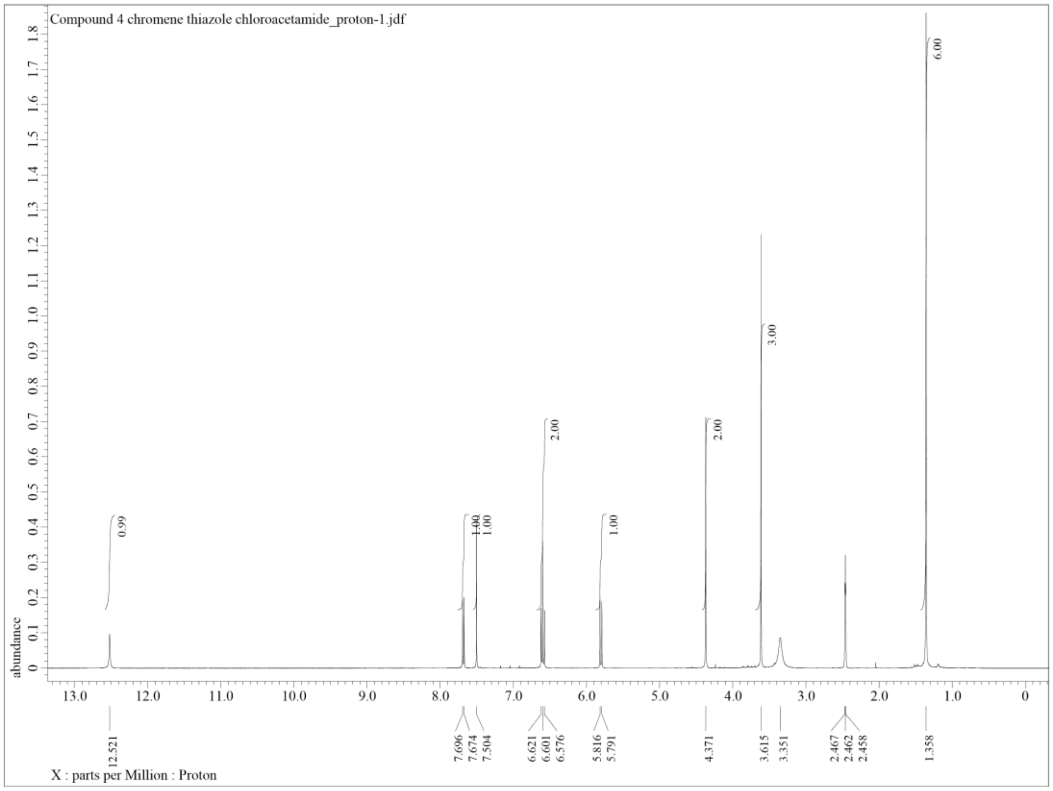

Figure S13. ^{1}H NMR spectrum for compound **4** in DMSO-d_6

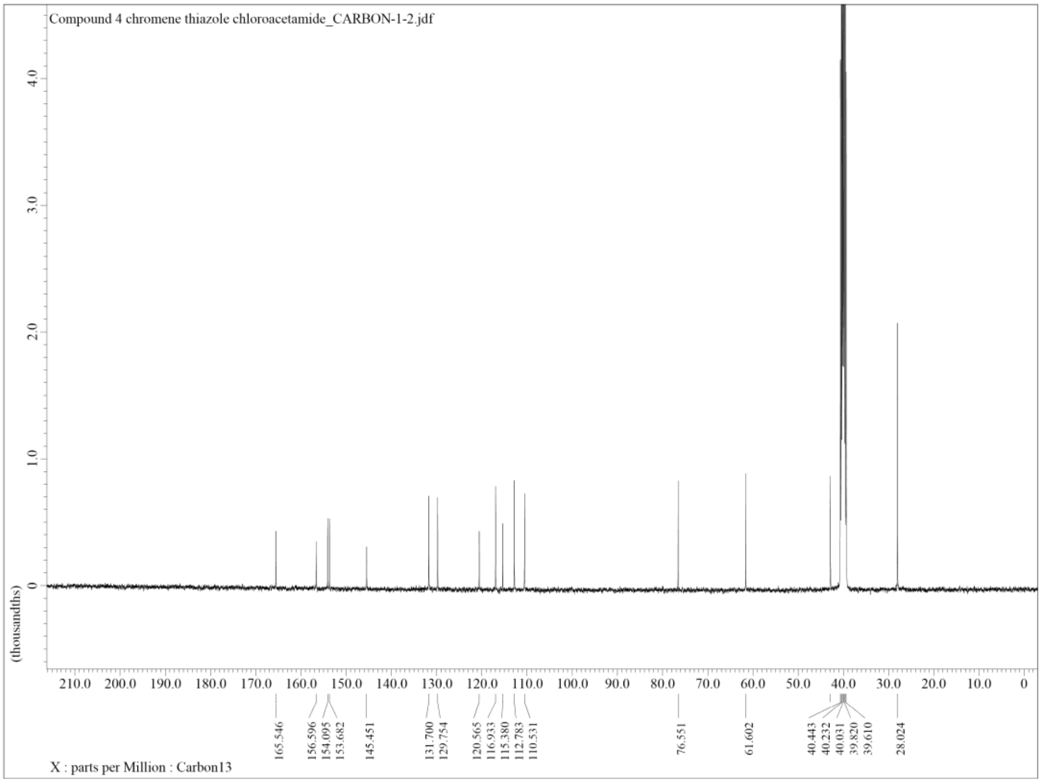

Figure S14. ^{13}C NMR spectrum for compound **4** in DMSO-d_6

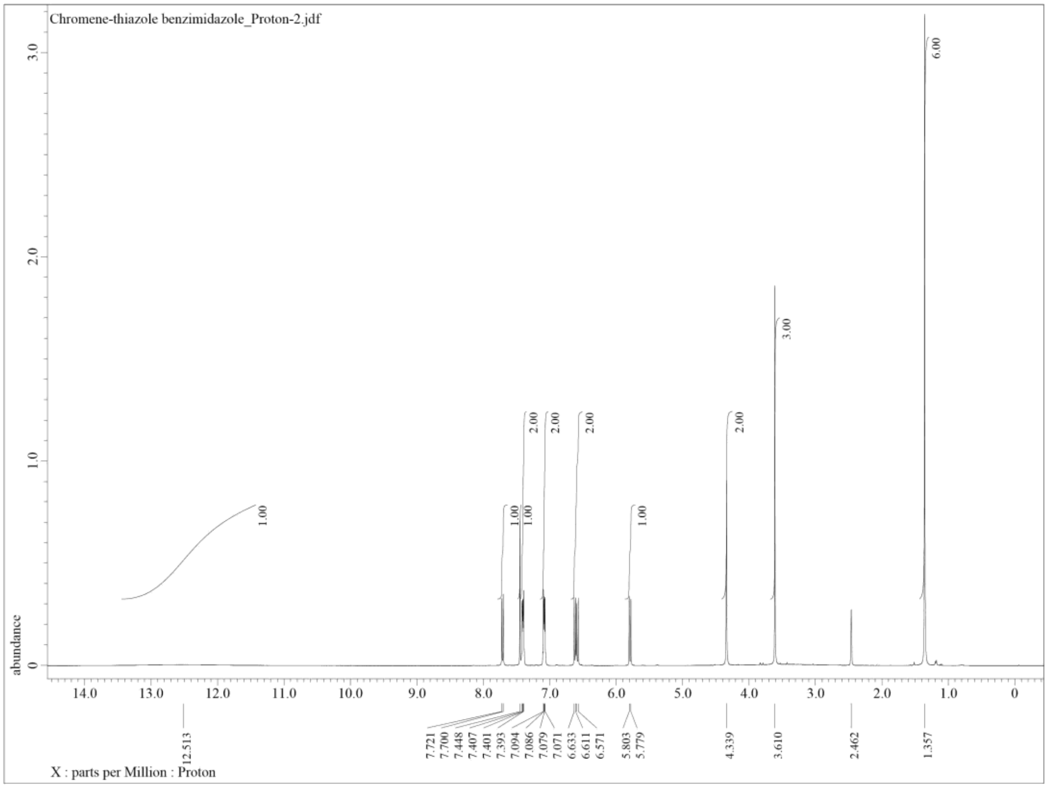

Figure S15. ^{1}H NMR spectrum for compound **5** in DMSO-d_6

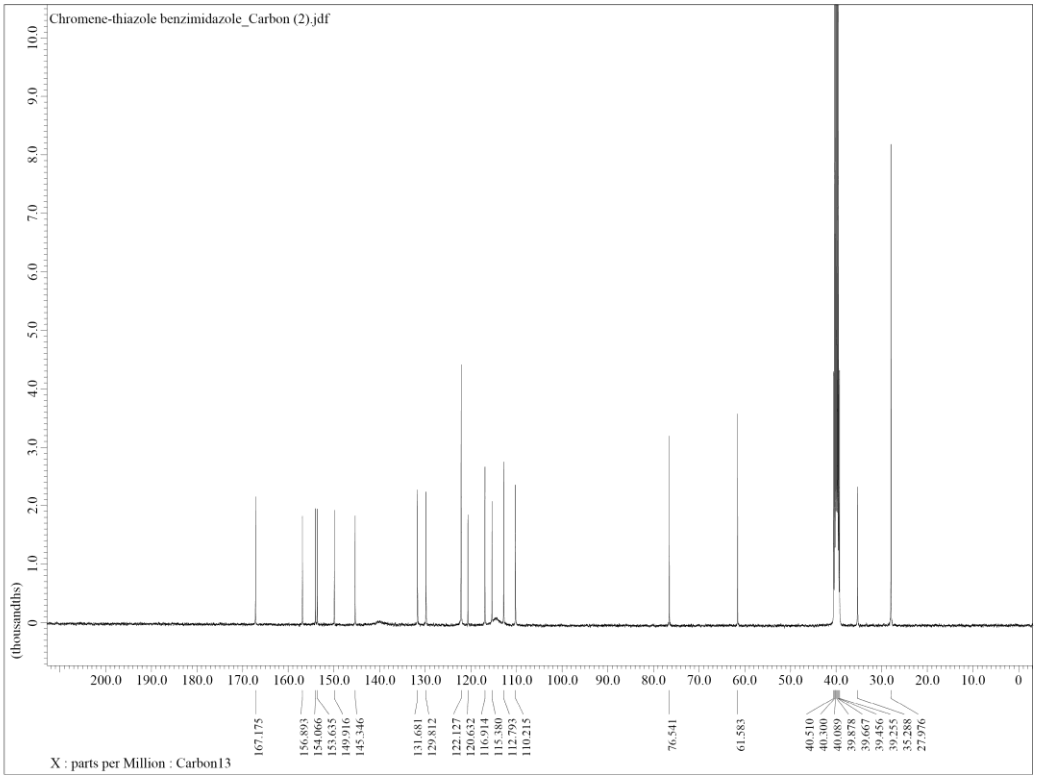

Figure S16. ^{13}C NMR spectrum for compound **5** in DMSO-d_6

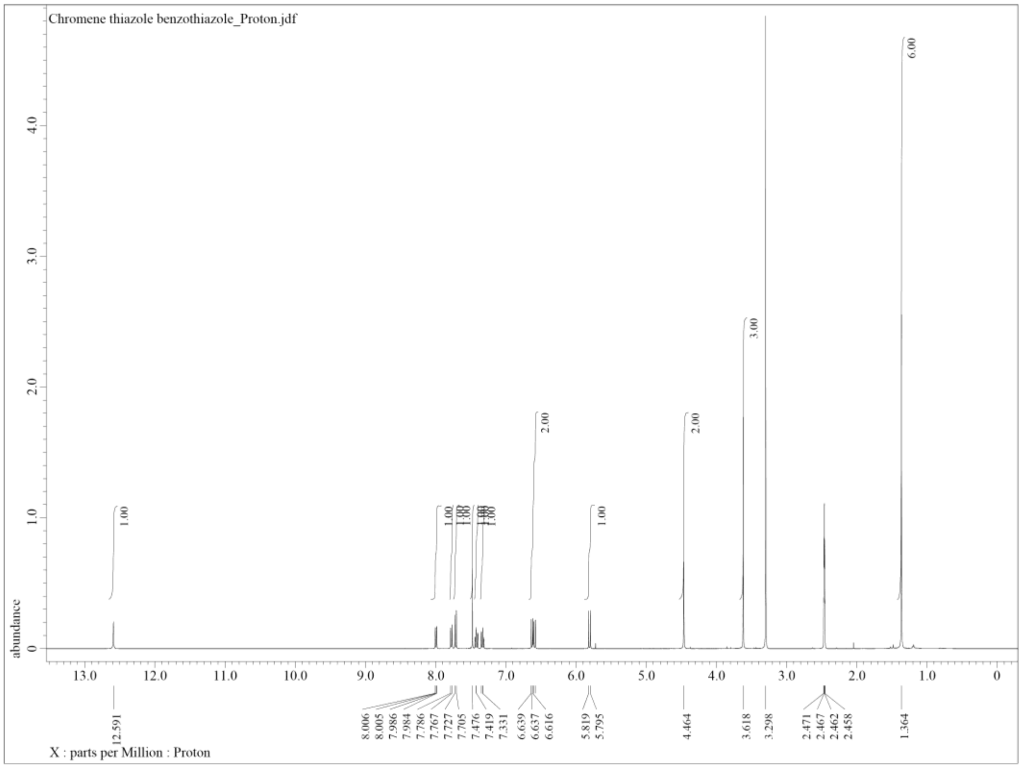

Figure S17. ^{1}H NMR spectrum for compound **6** in DMSO-d_6

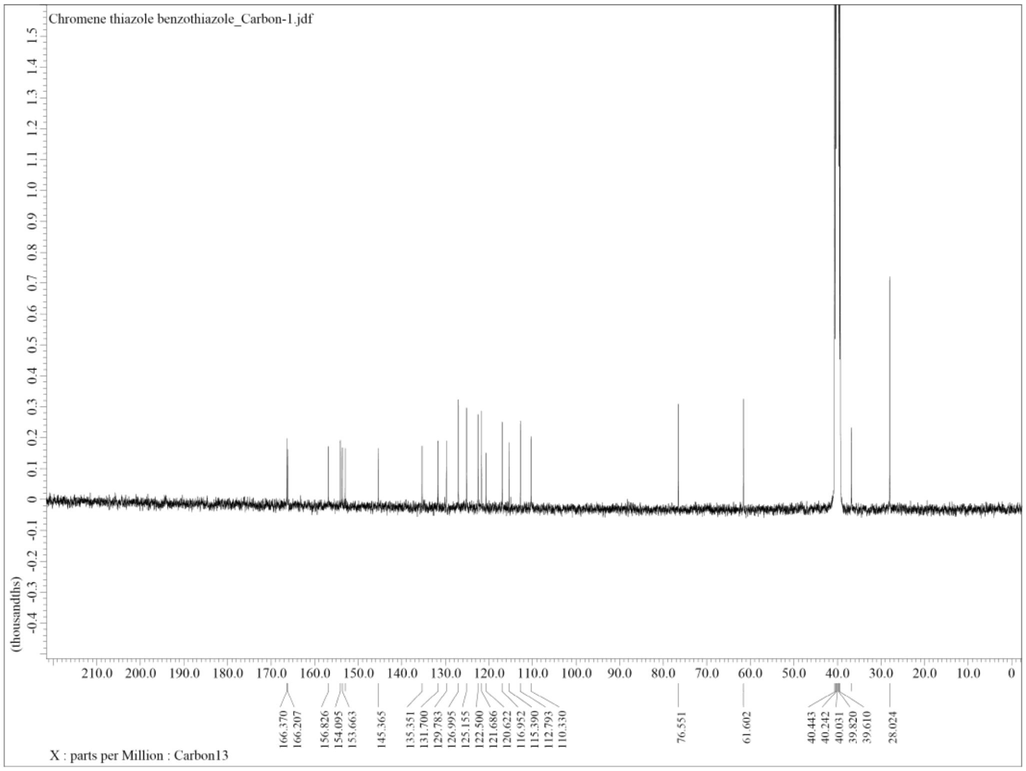

Figure S18. ^{13}C NMR spectrum for compound **6** in DMSO-d_6

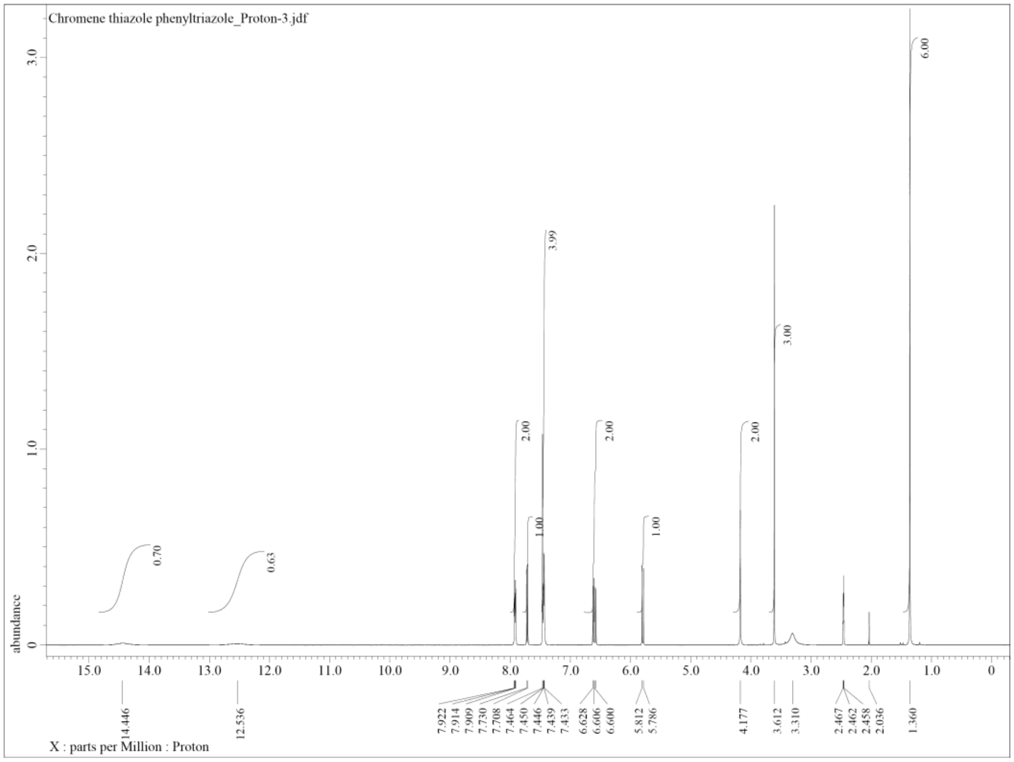

Figure S19. ^{1}H NMR spectrum for compound **7** in DMSO-d_6

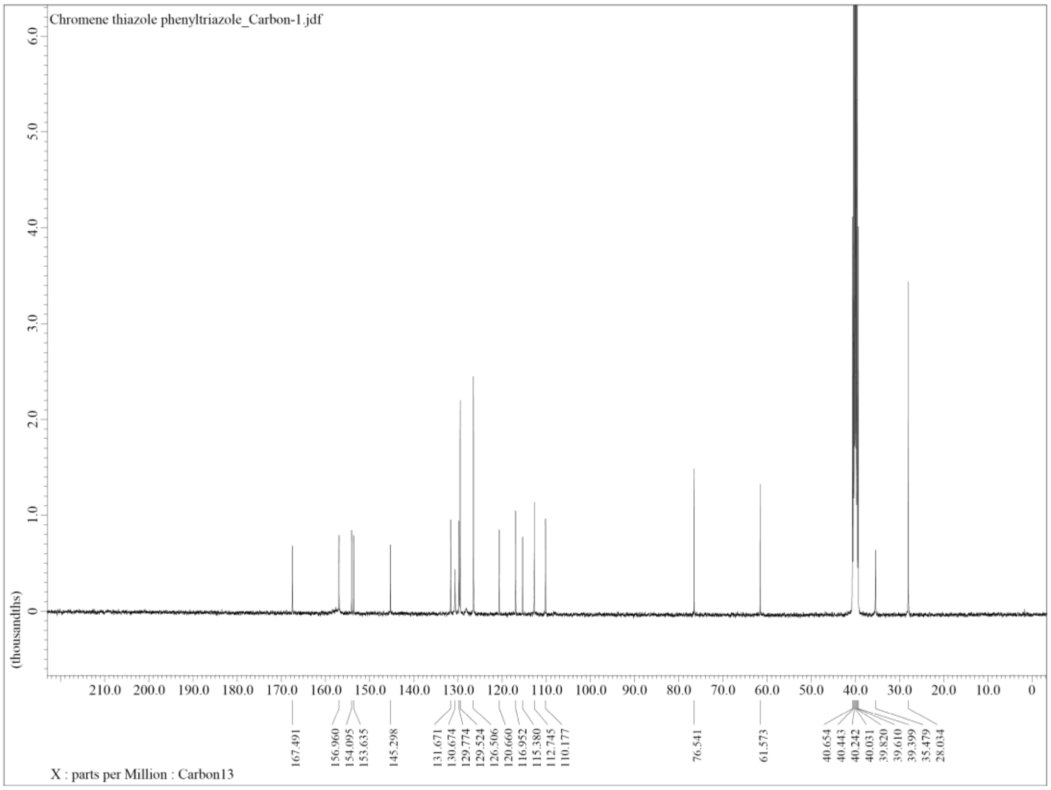

Figure S20. ^{13}C NMR spectrum for compound **7** in DMSO-d_6

Molecular Dynamics and QM Calculations

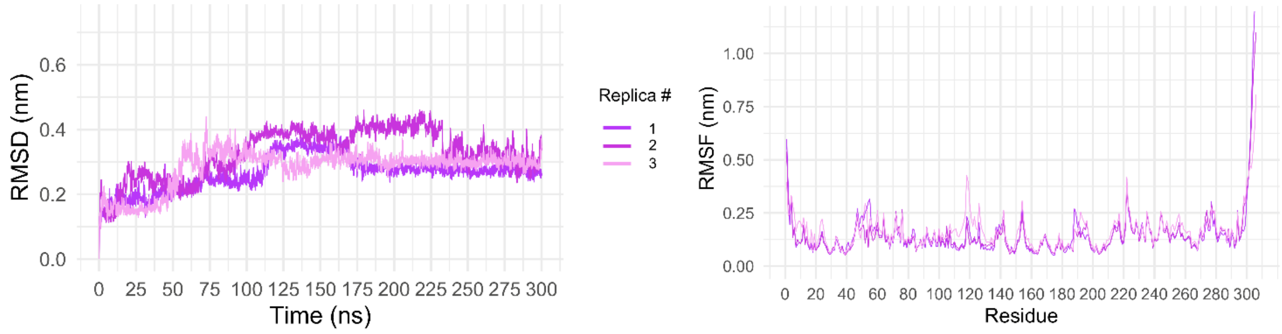

Figure S21. RMSD and RMSF of M^{pro} in complex with benzimidazole derivative (**5**)

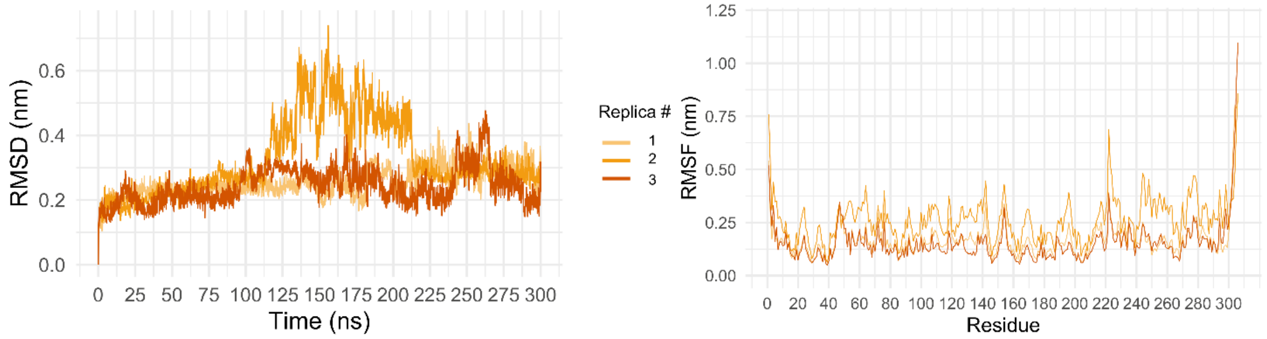

Figure S22. RMSD and RMSF of M^{pro} in complex with benzothiazole derivative (**6**)

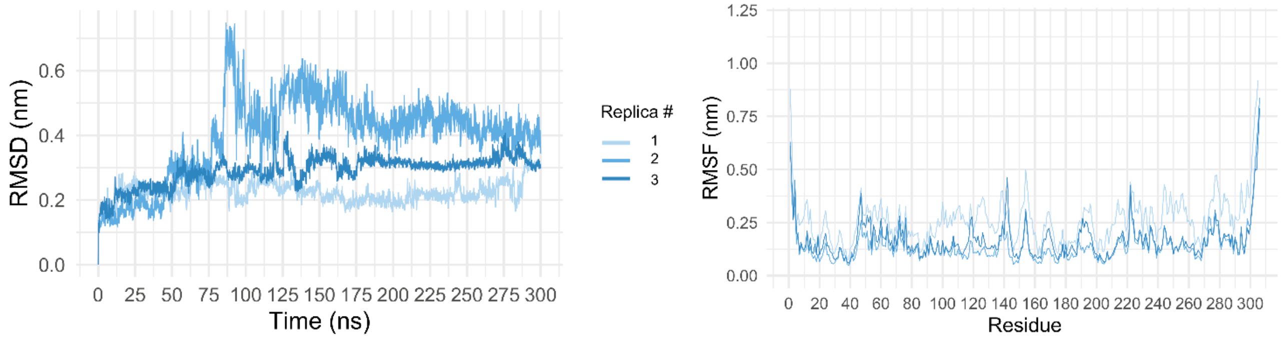

Figure S23. RMSD and RMSF of M^{pro} in complex with phenyltriazole derivative (**7**)

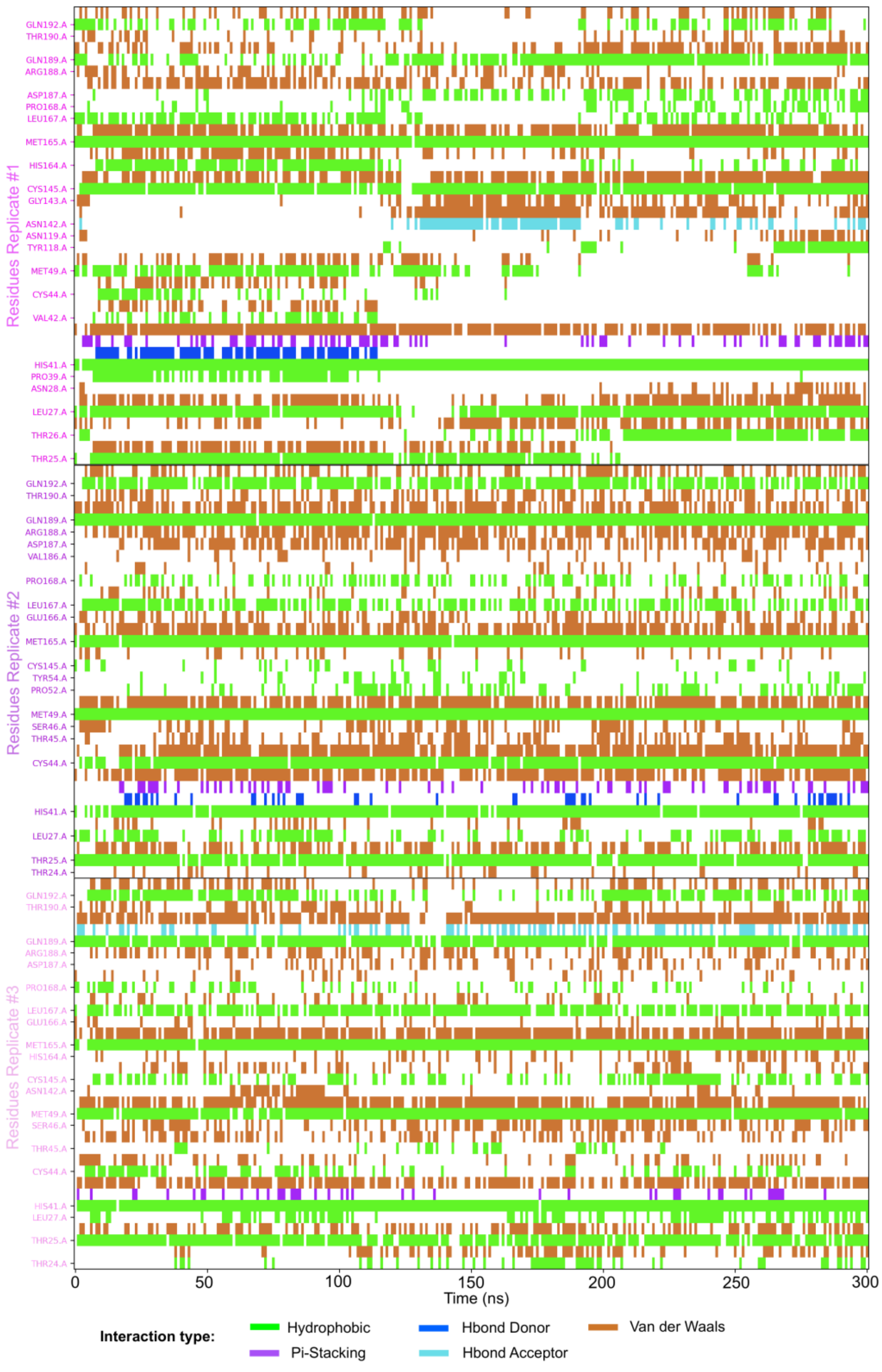

Figure S24. Analysis of interaction between M^{pro} and benzimidazole derivative (**5**) along 300 ns trajectory

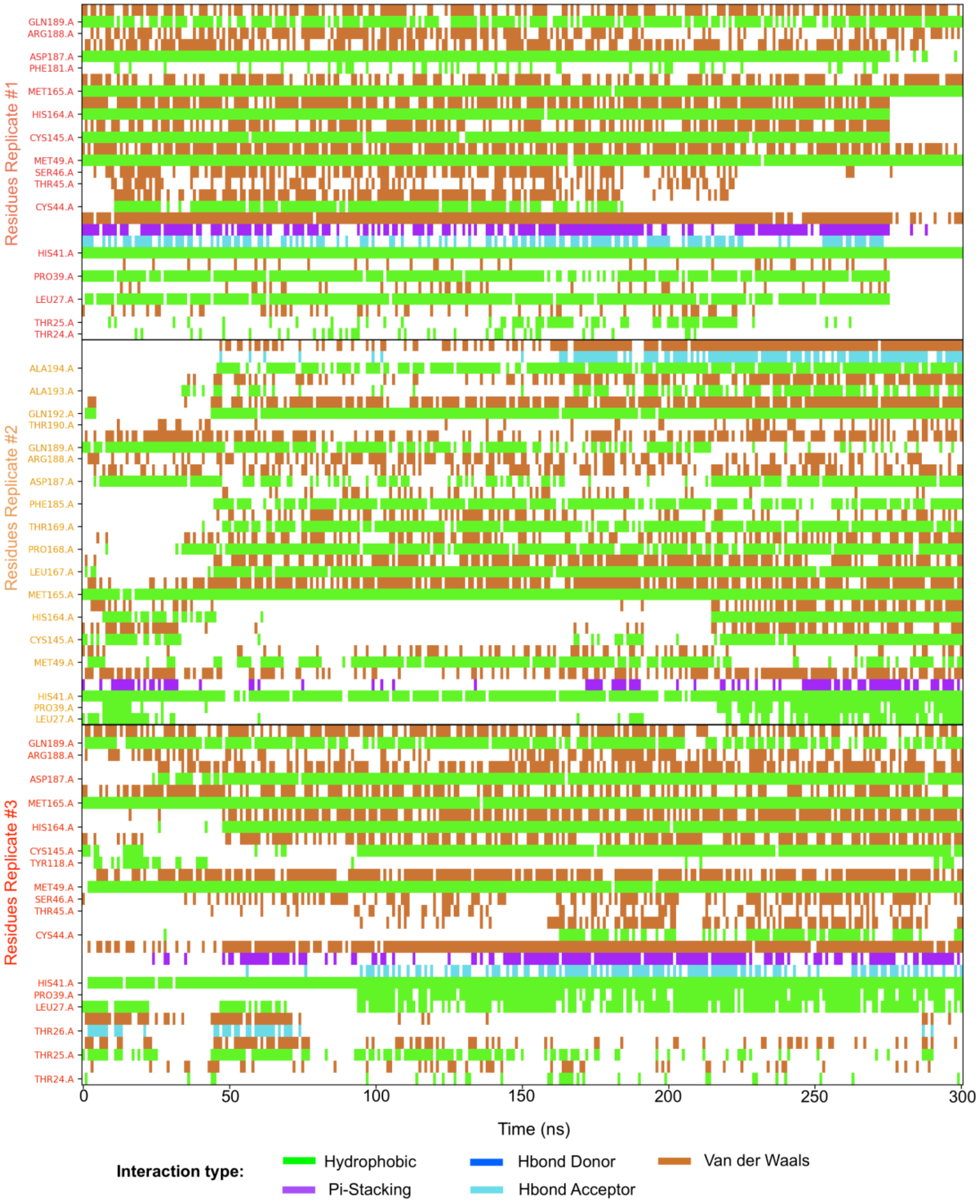

Figure S25. Analysis of interaction between M^{pro} and benzothiazole derivative (**6**) along 300 ns trajectory

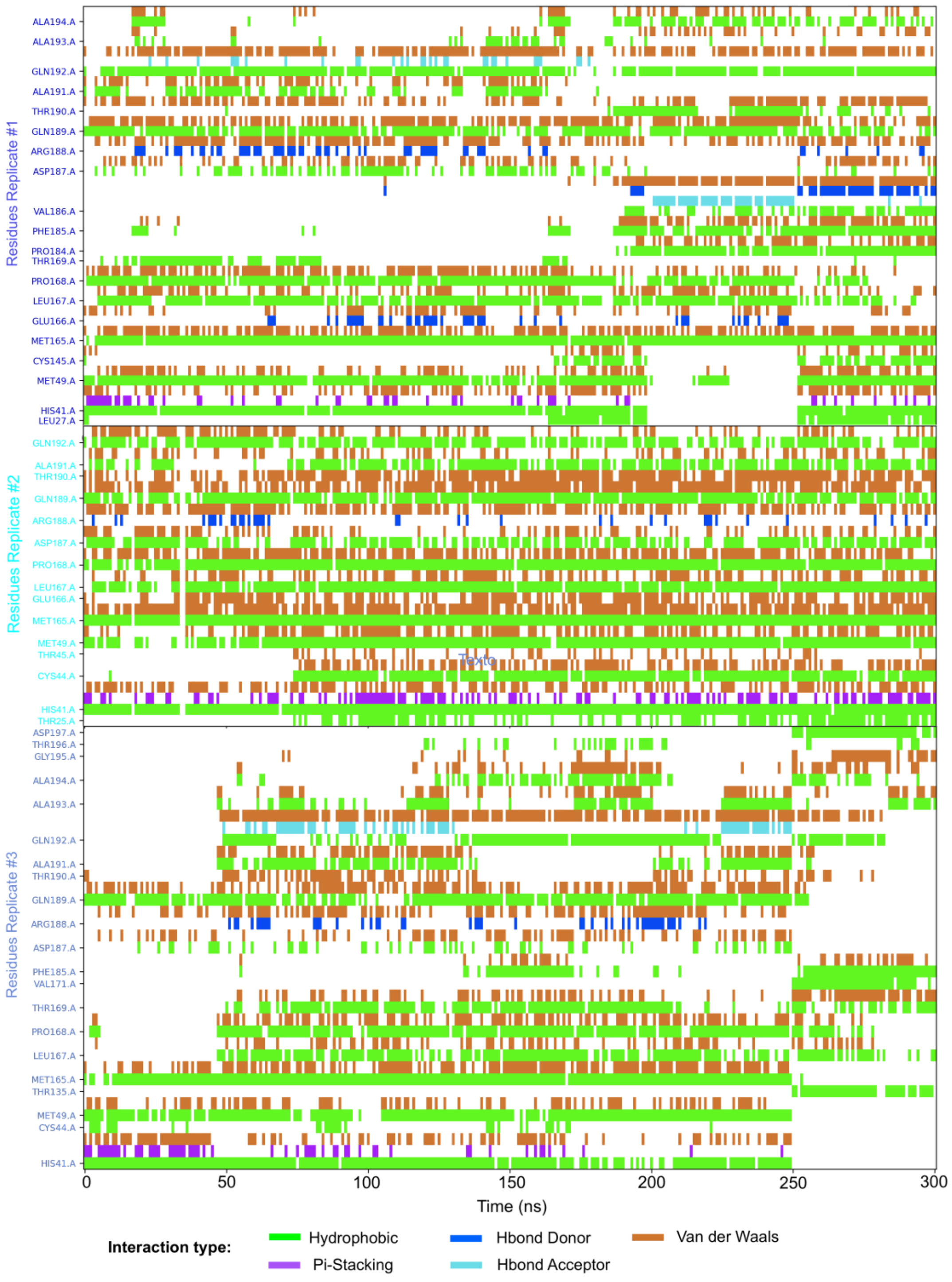

Figure S26. Analysis of interaction between M^{pro} and phenyltriazole derivative (**7**) along 300 ns trajectory

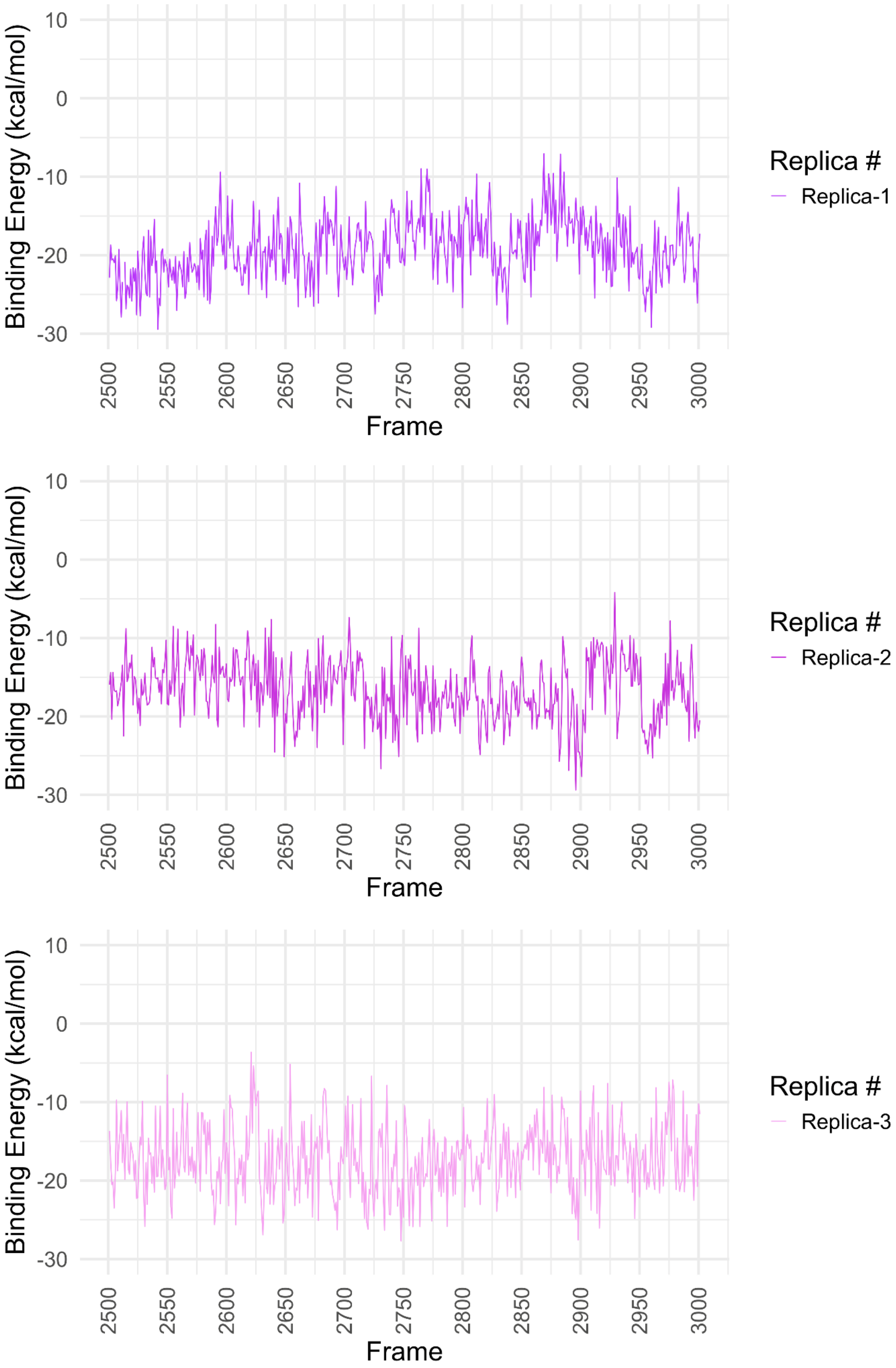

Figure S27. QM/MM plot for M^{pro} in complex with benzimidazole derivative (**5**)

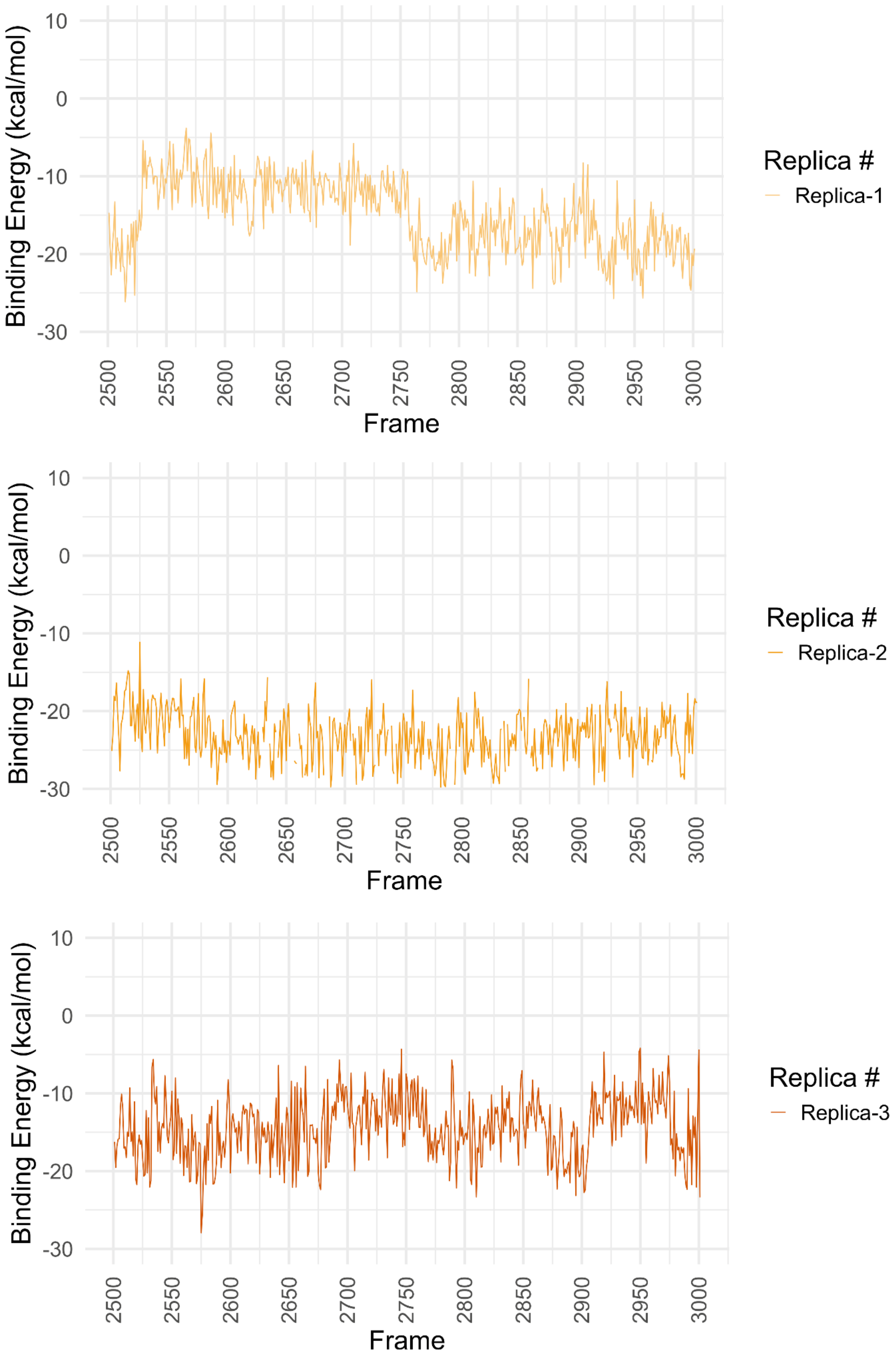

Figure S28. QM/MM plot for M^{pro} in complex with benzothiazole derivative (**6**)

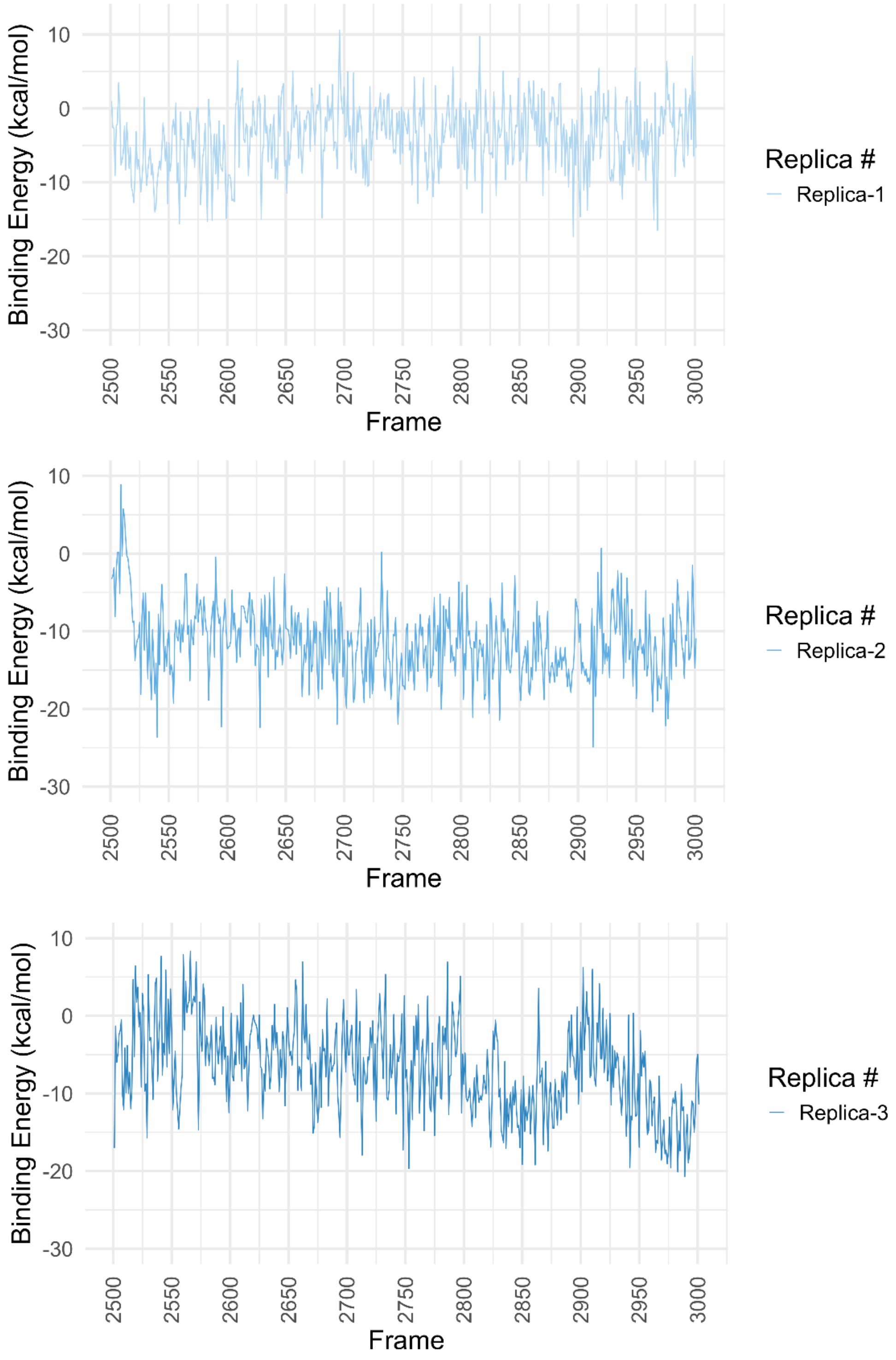

Figure S29. QM/MM plot for M^{pro} in complex with phenyltriazole derivative (**7**)
